# Proteomic Analysis of Plant-Derived hIGF-1-Fc Reveals Proteome Abundance Changes Associated with Wound Healing and Cell Proliferation

**DOI:** 10.3390/proteomes13040059

**Published:** 2025-11-07

**Authors:** Kittinop Kittirotruji, Utapin Ngaokrajang, Visarut Buranasudja, Ittichai Sujarittham, San Yoon Nwe, Pipob Suwanchaikasem, Kaewta Rattanapisit, Christine Joy I. Bulaon, Waranyoo Phoolcharoen

**Affiliations:** 1 Graduate Program of Program in Research for Enterprise, Chulalongkorn University, Bangkok 10330, Thailand; 6572011633@student.chula.ac.th; 2 BGF Plantrix Co., Ltd., Bangkok 10330, Thailand; utapin.n@baiyaphytopharm.com; 3 Department of Pharmacology and Physiology, Faculty of Pharmaceutical Sciences, Chulalongkorn University, Bangkok 10330, Thailand; visarut.b@pharm.chula.ac.th; 4 Center of Excellence in Natural Products for Ageing and Chronic Diseases, Chulalongkorn University, Bangkok 10330, Thailand; 5 Center of Excellence in Plant-produced Pharmaceuticals, Chulalongkorn University, Bangkok 10330, Thailand; grimm1999@hotmail.com; 6 Department of Pharmacognosy and Pharmaceutical Botany, Faculty of Pharmaceutical Sciences, Chulalongkorn University, Bangkok 10330, Thailand; 7 Department of Research and Development, Baiya Phytopharm Co., Ltd., Bangkok 10330, Thailand; sanyoon.n@baiyaphytopharm.com (S.Y.N.); pipob.s@baiyaphytopharm.com (P.S.); kaewta.r@baiyaphytopharm.com (K.R.)

**Keywords:** cell migration, cytoskeleton, hIGF-1-Fc, proteomics, ribosome

## Abstract

Background: Human insulin-like growth factor 1 (hIGF-1) plays a key role in cell proliferation and tissue repair. While plant expression systems offer a cost-effective and scalable alternative for recombinant protein production, the molecular effects of plant-derived hIGF-1 on mammalian cells remain largely unexplored. Methods: In this study, a recombinant fusion protein of hIGF-1 with human Fc (hIGF-1-Fc) was transiently expressed in *Nicotiana benthamiana* using the geminiviral pBYR2e system and purified by Protein A affinity chromatography. SDS-PAGE and Western blotting confirmed the predicted molecular weight, and LC-MS identified N-glycosylation at the Fc N229 site with plant-type glycans such as GnMXF, GnGnXF, and MMXF. Bioactivity was evaluated using MCF-7 cell proliferation and NIH3T3 wound healing assays. Label-free quantitative proteomics was performed on NIH3T3 fibroblasts to assess molecular changes. Results: hIGF-1 Fc significantly promoted cancer cell migration and fibroblast proliferation. Proteomic profiling revealed an abundance of cytoskeletal proteins such as actin and tubulin and metabolic enzymes related to energy production. Gene ontology and pathway enrichment analyses indicated significant modulation of ribosome biogenesis and carbon metabolism. Conclusions: This study presents the first proteome-level investigation of plant-produced hIGF-1-Fc in mouse fibroblasts and reveals its impact on cytoskeletal organization and metabolic pathways involved in proliferation and wound healing.

## 1. Introduction

Cellular agriculture represents an innovative approach to producing recombinant proteins or agricultural commodities using cell cultures derived from animals, plants, and microbes [1]. Central to this expression method are growth factors that regulate various cellular processes, including cell growth, tissue repair, and differentiation [2]. They are indispensable in the fields of stem cell research, cancer therapeutics, cell and gene therapy, and in pharmaceutical and cosmetic products. However, their high production costs limit their accessibility, as growth factors account for up to 90% of expenses in the cellular agriculture industry [3].

Among growth factors, human insulin-like growth factors (hIGFs), fibroblast growth factor (FGF), and epidermal growth factor (EGF) are particularly important [4]. hIGF comprises two types—hIGF-1 and hIGF-2 subtypes—and it plays a pivotal role in cell proliferation, survival, and differentiation [5]. This 7.6 kDa protein consists of 70 amino acids with disulfide-linked A and B chains and is primarily secreted by the liver, where it synergizes with growth hormone to mediate anabolic and growth-promoting effects [6]. Recently, hIGF-1 has become an essential supplement in cell culture media formulations, particularly for enhancing growth rates and skeletal muscle development [4]. Additionally, its therapeutic potential in wound healing has been well documented in the literature. For example, hIGF-1 promotes cell migration, proliferation, and extracellular matrix production, all of which are crucial for effective wound repair [7].

Plant molecular pharming has emerged as a promising platform that employs plants as bioreactors to manufacture target proteins in a scalable and economical manner. Plants from the *Nicotiana* genus are commonly used hosts due to their rapid growth period and ease of genetic manipulation, though other crops like tomato, rice, lettuce, or potato are also viable options. Advantages of this platform include simplified cultivation, a low pathogen contamination risk, rapid mass protein production, scalability, and the ability to produce complex proteins [8]. While plant-based expression of growth factors, e.g., human vascular endothelial growth factor (VEGF) [9], EGF [10], and FGF [11], has been explored, few studies have investigated their functional effects at the molecular level.

Proteomics is a powerful approach to study growth-factor-induced cellular responses. Previous studies have analyzed differential proteome changes and pathway modulation following growth factor treatment [12,13]. For instance, Nagano et al. analyzed the proteomic changes in Swiss 3T3 fibroblasts after long-term exposure to platelet-derived growth factor (PDGF), IGF-1, and EGF [12]. Their findings revealed that PDGF induced the most sustained phospho-signaling and protein synthesis, whereas IGF-1 and EGF contributed to distinct proteomic profiles affecting key regulators of actin cytoskeleton remodeling, translation, and chaperonin activity. Similarly, another research evaluated the acute effects of IGF-1 on murine C2C12 myoblasts, identifying 23 increased and 17 decreased proteins following IGF-1 treatment [13]. These results highlight the impact of IGF-1 on metabolic and structural protein networks in muscle cells. However, to date, no studies have systematically analyzed the proteomic response of fibroblasts to plant-produced hIGF-1.

In this study, we conducted the first proteome-wide investigation of plant-made hIGF-1-Fc in mouse NIH3T3 fibroblasts. The recombinant hIGF-1 was fused with an Fc fragment and expressed in *N. benthamiana* tobacco plants. The fusion protein was purified via Protein A affinity chromatography, and the purified hIGF-1-Fc was subsequently evaluated for its effects on cell proliferation and wound healing in mammalian cell lines. Additionally, quantitative proteomics was performed to identify differentially expressed proteins in response to hIGF-1-Fc treatment.

## 2. Materials and Methods

### 2.1. Host, Expression Vector, and Cell Line

Wildtype *N. benthamiana* seeds were kindly gifted by Dr. Supaart Sirikantaramas (Faculty of Science, Chulalongkorn University, Bangkok, Thailand). Competent cells, *Escherichia coli* DH10B (Goldbio, CC-100-5x50) and *Agrobacterium tumefaciens* GV3101 (Goldbio, CC-105-5x50), were used for subcloning. The pBYR2eK2Md or pBYR2e vector [14,15] was utilized for recombinant protein expression in plants. Antibiotics used for clone selection included kanamycin (Bio Basic, Markham, ON, Canada), rifampicin (Thermo Fischer Scientific, Waltham, MA, USA), and gentamicin (ITW Reagents, Darmstadt, Germany). Restriction enzymes and the T4 ligation kit were purchased from New England Biolabs (Ipswich, MA, USA), and plasmid purification was performed using a DNA-spin™ kit (iNtRON Biotechnology, Seongnam-si, Gyeonggi-do, South Korea). Plasmid DNAs were sent for Sanger sequencing analysis to U2Bio, Thailand. Protein all-blue standards (Bio-Rad Laboratories, Hercules, CA, USA), InstantBlue^®^ Coomassie protein stain (Abcam plc, Cambridge, UK), and goat anti-human IgG-HRP antibody (SouthernBiotech, Birmingham, AL, USA) were used for protein analysis and detection. Mouse embryonic fibroblasts (NIH3T3) and a human breast cancer cell line (MCF-7) were provided by Prof. Jittima Luckanagul (Faculty of Pharmaceutical Sciences, Chulalongkorn University), originally sourced from ATCC (Manassas, VA, USA). All other reagents, DMEM (Biowest, Riverside, MO, USA), FBS (HyClone, Logan, UT, USA), penicillin–streptomycin (Gibco, Waltham, MA, USA), L-glutamine (Gibco), and MTT (Invitrogen, Carlsbad, CA, USA), were procured for cell-based assays.

### 2.2. Plasmid Construction

**Figure 1:**
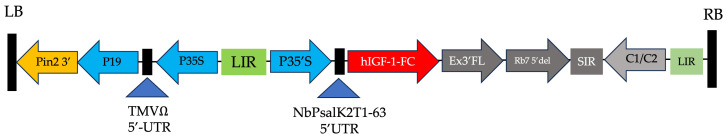
Schematic of the geminiviral vector containing the hIGF-1-Fc gene. Key elements include the T-DNA left and right borders (LB and RB) for gene insert integration into plant cells, the CaMV 35S promoter (P35S) enhanced by TEV 5′ for translation, and P19 for gene silencing suppression. Additional regions for mRNA stability, replication, expression, and termination include the LIR and SIR of the bean yellow dwarf virus (BeYDV) and BeYDV ORFs C1 and C2. Diagram adapted from previous work on plant-produced growth factors [9,10].

The hIGF-1 gene (Uniprot No: P05019, G49-A118) was constructed as a fusion protein with a human IgG Fc fragment at the C-terminus and flanked by XbaI/SacI restriction sites. The hIGF-1-Fc codon sequence was optimized for expression in *N. benthamiana* and synthesized by Genewiz (Genewiz, Suzhou, China). The optimized gene was then subcloned into the XbaI and SacI sites of the geminiviral vector pBYR2e to generate the pBYR2e/hIGF-1-Fc plasmid (Figure 1). The ligated plasmid was introduced into *E. coli* DH10B competent cells by heat shock and cultured on LB agar containing 50 mg/L kanamycin overnight at 37 °C. Transformants were screened by PCR, and positive clones were grown in LB broth with 50 mg/L kanamycin. The presence of the hIGF-1-Fc insert was confirmed by DNA sequencing (Supplementary Figure S1).

### 2.3. Transient Expression of hIGF-1 Fusion Protein

Production of recombinant hIGF-1-Fc in tobacco plants was conducted as previously described [11]. The pBYR2e/hIGF-1-Fc vector was transformed into competent *A. tumefaciens* GV3101 via electroporation. Transformants were selected on LB agar plates containing 50 mg/L kanamycin, 50 mg/L rifampicin, and 50 mg/L gentamicin and incubated for 48 h at 28 °C. Positive colonies were then cultured in LB broth with the same antibiotics overnight at 28 °C. After reaching an OD600 of 0.2, *Agrobacterium* harboring hIGF-1-Fc gene was infiltrated into four-week-old *N. benthamiana* plants. Leaves were harvested at 1, 3, 5, and 7 days post-infiltration (dpi) to optimize protein expression levels. Expressed protein abundance was assessed by Western blotting, and band intensity was quantified by ImageJ (version 1.52p, National Institutes of Health, Bethesda, MD, USA) for comparison. The membrane was probed using an HRP-conjugated anti-human IgG Fc antibody to specifically detect the hIGF-1-Fc protein.

### 2.4. Affinity-Based Purification of Plant-Derived hIGF-1 Fusion Protein

The affinity chromatography purification procedure was adapted from [16]. Approximately 0.25 kg of plant leaves was homogenized in ice-cold PBS buffer pH 7.4 at a 1:2. The crude extract was centrifuged at 15,000 rpm for 45 min at 4 °C, followed by filtration using a 0.45 µm S-Pak membrane filter (Merck, Burlington, MA, USA). The clear supernatant containing the expressed hIGF-1-Fc protein was loaded onto a Protein A gravity-flow chromatography column (Cytiva, Marlborough, MA, USA). The column was washed with PBS buffer pH 7.4, and the bound fusion protein was eluted using 0.1 M glycine buffer pH 2.7. The eluted fraction was subsequently neutralized with 1.5 M Tris-HCl buffer pH 8.8 to reach pH 7.4. The yield of hIGF-1-Fc was measured using a Bradford assay and human IGF-1 ELISA kit (R&D systems, Minneapolis, MN, USA, DY291).

### 2.5. SDS-PAGE and Western Blotting

Crude extracts and purified hIGF-1-Fc were analyzed by SDS-PAGE and Western blotting, as previously described [17]. Equal amounts of total soluble proteins (6 µg) from leaves harvested at 1, 3, 5, and 7 dpi and 2 µg of pure hIGF-1-Fc were mixed with SDS loading buffer, boiled for 5 min at 95 °C, and separated on 12% SDS-PAGE gels under both non-reducing and reducing conditions. Parallel acrylamide gels were resolved under identical conditions: one was stained with InstantBlue^®^ Coomassie protein stain following the manufacturer’s instructions, while the other was subjected to Western blot analysis. Proteins were transferred onto a nitrocellulose membrane using a wet-transfer system with 1X transfer buffer (25 mM Tris, 192 mM glycine, 15% methanol) operated at 100 V for 90 min at 4 °C. The blots were subsequently blocked with 5% (*w*/*v*) skimmed milk in PBS buffer pH 7.4 for 1 h at room temperature and incubated with an HRP-linked goat monoclonal antibody specific to human IgG Fc (SouthernBiotech) at a 1:5000 dilution for 2 h at room temperature with gentle agitation. The target protein bands were detected using a chemiluminescence detection system. Band intensities were quantified using ImageJ (version 1.52p) and reported as raw values without normalization to housekeeping protein.

### 2.6. LC-MS Glycoprofiling

Protein solution with approximately 15 µg of protein was reduced with 10 mM dithiothreitol (DTT) at 65 °C for 30 min and alkylated with 25 mM iodoacetamide (IAA) at room temperature for 20 min under darkness. Proteins were digested with 0.2 µg of trypsin at 37 °C for 4 h. The reaction was stopped with 1% FA. The solution was then centrifuged at 14,000 rpm for 10 min and transferred to LC-MS vial.

Peptides were analyzed using an Agilent 1290 Infinity II LC system coupled with an Agilent 6545XT Q-TOF mass spectrometer. LC separation was conducted on an AdvanceBio Peptide Mapping column (120 Å, 2.1 × 150 mm, 2.7 µm) at 60 °C. Injection volume was 10 µL. Mobile phase A was 0.1% FA in water, and mobile phase B was 0.1% FA in acetonitrile. LC gradient was set as follows: 0% B for 2 min, 0% to 20% B in 33 min, 20% to 30% B in 20 min, 30% to 50% B in 10 min, 50% to 90% B in 5 min, 90% B for 5 min, 90% to 0% B in 5 min, and 0% B for 5 min, with a constant flow rate of 0.4 mL/min. MS analysis was conducted in positive mode with MS and MS/MS mass range of 100–1700 and 50–1700 *m*/*z*, respectively. Acquisition times were 5 and 3 spectra per s for MS and MS/MS, respectively. MS parameters were set as follows: gas temperature at 325 °C, nebulizer at 35 psi, dying gas at 13 L/min, sheath gas temperature at 275 °C, sheath gas flow at 12 L/min, capillary voltage at 4000 V, nozzle voltage at 500 V, and skimmer voltage at 65 V. The top 10 precursor ions per cycle were selected for MS/MS fragmentation. The absolute precursor threshold was 3000 counts. Collision energy (CE) was varied according to the charge state of the peptide. For peptides with charge +1 and +2, the CE was calculated using a formula of (3.1 × ((*m*/*z*)/100) + 1), while for peptides with charge ≥+3, the CE was calculated using a formula of (3.6 × ((*m*/*z*)/100) − 4.8). Reference mass was monitored at 922.0098 *m*/*z*. Data was collected in centroid mode.

### 2.7. In Vitro Cell Proliferation Assay of Plant-Derived hIGF-1 Fusion Protein

MCF-7 cells were cultured in DMEM supplemented with 10% FBS, 1% penicillin–streptomycin, and 1% L-glutamine. Cells were seeded into a 96-well plate with a density of 5 × 10^3^ cells/well in DMEM and incubated overnight at 37 °C under a humified atmosphere with 5% CO_2_. An eight-point, ten-fold serial dilution of plant-produced hIGF-1-Fc or Fc, starting from a concentration of 10 µg/mL, was added to the cells. Untreated cells served as the control. After 48 h of treatment, 100 μL of MTT solution (0.50 mg/mL) was added and incubated for 4 h at 37 °C. The solution was carefully removed. Formazan crystals were dissolved by adding 100 µL of DMSO per well. Absorbance was measured at 570 nm using a microplate reader (CLARIOStar, BMG Labtech, Ortenberg, Germany). Cell viability was calculated as a percentage relative to the untreated control cells and represented as dose–response curves. EC_50_ values were calculated using GraphPad Prism 9.0. The assay was performed following prior MTT protocols [9].

### 2.8. In Vitro Wound Healing Assay of Plant-Derived hIGF-1 Fusion Protein

The wound healing efficacy of plant-produced hIGF-1-Fc was assessed using an in vitro scratch healing assay based on the method described by [18]. NIH3T3 fibroblasts were seeded at a density of 3 × 10^4^ cells/well. Once a confluent cell monolayer was achieved, a “wound” was created by scratching the cell layer with a sterile 200 µL pipette tip. The wells were washed twice with PBS buffer at pH 7.4 to remove any dislodged cells. Plant-produced hIGF-1-Fc was added to the culture medium at final concentrations of 50 ng/mL and 100 ng/mL, and cells were incubated for 24 h and 48 h. Wound areas were photographed using a phase-contrast microscope (Nikon Corporation, Tokyo, Japan) at 100X magnification. Microscopic images were captured at 0, 24, and 48 h using a Nikon Eclipse Ti-S microscope (Nikon Corporation) equipped with a DS-Ri2 camera and the NIS Element v5.01 software. The ImageJ software version 1.52p was used to measure the wound area and calculate the percentage of wound closure relative to the initial area at 0 h.

### 2.9. Preparation of Fibroblast Cell Lysates for Proteomics Analysis

NIH3T3 fibroblast cells were seeded into a cell culture dish at a density of 5 × 10^3^ cells/well, then treated with 50 and 100 ng/mL of plant-produced hIGF-1-Fc for 72 h. Cell pellets were collected and extracted with 100 µL of 50 mM ammonium bicarbonate (ABC) buffer with a metal bead in a Mixer Mill 400 (Retsch GmbH, Haan, Germany), following the method and protocol in the shotgun proteomics method and protocol book [19]. The samples were centrifuged at 14,000 rpm for 5 min, and the supernatant was collected. Protein concentration was measured with a Bradford assay and normalized to 2.3 mg/mL protein. The protein solution underwent treatment with 10 mM dithiothreitol (DTT) at 65 °C for 30 min to break disulfide bonds, followed by modification with 25 mM iodoacetamide (IAA) at room temperature for 20 min under darkness. Subsequently, proteins were enzymatically cleaved using 0.5 µg of trypsin at 37 °C for 4 h. To quench the reaction, 10% formic acid (FA) was added, and the mixture was centrifuged at 14,000 rpm for 10 min. The clarified supernatant was transferred into a polypropylene vial and subjected to LC-MS/MS analysis.

### 2.10. Proteomics LC-MS/MS Acquisition

Peptide samples were analyzed using Agilent 1290 Infinity II LC system coupled with an Agilent 6545XT Q-TOF mass spectrometer. Chromatographic separation was performed on an AdvanceBio Peptide Mapping column (120 Å, 2.1 × 150 mm, 2.7 µm) maintained at 60 °C. For each analysis, 10 µL of peptide solution was injected. The mobile phase consisted of 0.1% formic acid in water (mobile phase A) and 0.1% formic acid in acetonitrile (mobile phase B). The gradient elution protocol began with an isocratic hold at 0% B for 2 min, followed by a linear increase to 20% B in 33 min, then to 30% B over the subsequent 20 min. The gradient was further increased to 50% in 10 min, then to 90% B over 5 min, maintained at 90% B for 5 min, reduced back to 0% B in 5 min, and finally equilibrated at 0% B for 5 min. The flow rate was set at 0.4 mL/min throughout the run. Mass spectrometry was operated in positive mode with a mass range of 100–1700 *m*/*z*. Instrument settings included a gas temperature at 325 °C, nebulizer pressure at 35 psi, dying gas flow at 13 L/min, sheath gas temperature at 350 °C with a flow rate of 12 L/min, capillary voltage at 4000 V, nozzle voltage at 500 V, fragmentor voltage at 175 V, and skimmer voltage at 65 V. Data acquisition was performed at a rate of one spectrum per second. During MS/MS acquisition, up to two precursor ions were selected per cycle for fragmentation. Collision energy (CE) was adjusted according to the peptide charge state. For peptides with charge +1 and +2, the CE was calculated using a formula of (3.1 × (*m*/*z*)/100) + 1), while peptides with charge +3 or greater, the CE was calculated as (3.6 × (*m*/*z*)/100) − 4.8).

### 2.11. Proteomics Data Analysis and Pathway Analysis

Raw Agilent .d files were converted to .mzML files using the msConvert command in the ProteoWizard software [20] with default settings. Then, the .mzML file was converted to a .mzxML file using the TOPPAS function in the OpenMS software [21]. Data files in .mzxML format were loaded into the MaxQuant software version 2.4 [22]. All default parameters for the Agilent QTOF instrument and LFQ function were applied. Trypsin was selected as a digestive enzyme, and two missed cleavages were allowed. Methionine oxidation and N-terminal acetylation were set as variable modifications, while cysteine carbamidomethylation was a fixed modification. Protein and peptide match was searched with a high confidence level at 0.01 FDR. The mouse protein database was acquired from the UniProt website [23]. Proteins identified by site and reverse peptides were removed. LFQ intensity was used for statistical analysis using MetaboAnalyst version 6.0 [24] (https://www.metaboanalyst.ca/ accessed on 1 July 2025). Data was log 10 transformed and mean-centered.

The Kyoto Encyclopedia of Genes and Genomes (KEGG) [25] and gene ontology (GO) pathway analyses were utilized to identify the possible mechanisms underlying its effects via the online bioinformatics tool [26] (https://www.bioinformatics.com.cn/ accessed on 1 July 2025).

### 2.12. Statistical Analysis

The day post-infiltration optimization experiment was conducted with three biological replicates. For all subsequent characterization and bioactivity assays, a single large-scale batch of purified hIGF-1-Fc was used to ensure consistency across all tests. Functional assays were performed in triplicate using this protein batch, and data are presented as mean ± standard deviation (SD). An unpaired *t*-test was conducted to assess differences between individual groups. Additionally, two-way analyses of variance (ANOVA) were performed using the GraphPad Prism 9.0 software (San Diego, CA, USA). A *p*-value of <0.05 was considered statistically significant.

## 3. Results

### 3.1. Effects of Dpi on hIGF-1-Fc Protein Expression

**Figure 2:**
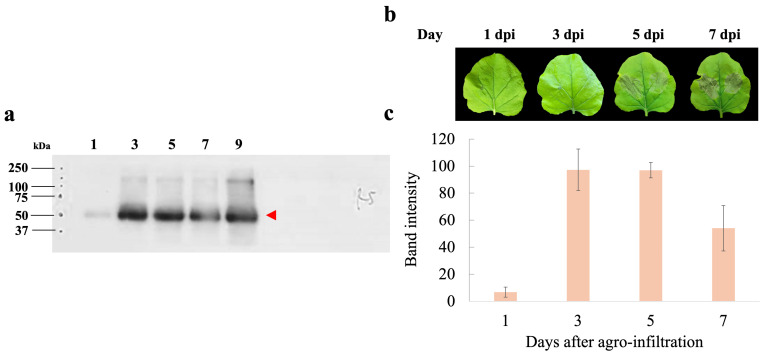
Days post-infiltration (dpi) optimization of hIGF-1-Fc production in *N. benthamiana*. (**a**) Western blotting of crude leaf extracts collected at 1, 3, 5, and 7 days after infiltration. The red arrow indicates the molecular weight of the target protein (approximately 70 kDa). (**b**) Representative images of leaf phenotypes at different dpi. (**c**) Bar graph depicting the measured band intensity values from the immunoblots.

To assess the expression levels of hIGF-1-Fc, infiltrated tobacco leaves were harvested at different dpi (1, 3, 5, and 7 days). The band intensity of the target protein increased over time post-infiltration. Western blot analysis demonstrated that transient expression of hIGF-1-Fc peaked at 3 dpi (Figure 2a), with the corresponding uncropped blots provided in Supplementary Figure S2. Expression levels remained relatively stable up to 5 dpi, followed by a decline at 7 dpi (Supplementary Table S1). The lowest protein expression level was observed at 1 dpi. Morphological assessments of the infiltrated leaf revealed minimal necrosis at 1 and 3 dpi, whereas visible necrotic symptoms appeared at 5 and 7 dpi (Figure 2b). Quantitative analysis of hIGF-1-Fc protein band intensity in crude supernatants showed values of 6.80 at 1 dpi, 97.29 at 3 dpi, 97.00 at 5 dpi, and 54.08 at 7 dpi (Figure 2c). Based on these results, the optimal harvest time for recombinant hIGF-1-Fc production was determined to be 3 dpi.

### 3.2. Purification of Plant-Produced hIGF-1-Fc

**Figure 3:**
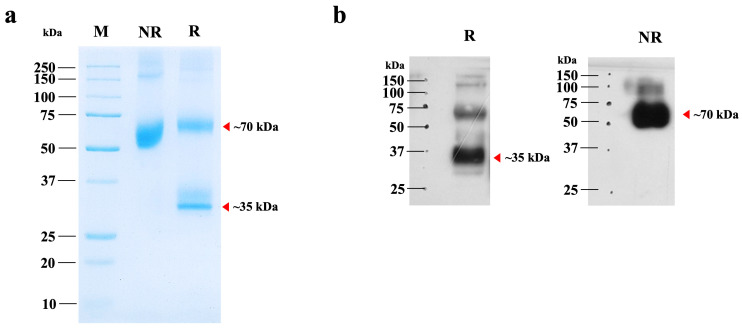
SDS-PAGE and Western blot analyses of purified hIGF-1-Fc from *N. benthamiana*. (**a**) SDS-PAGE analysis of 2 µg of purified hIGF-1-Fc under non-reducing (NR) and reducing (R) conditions. (**b**) Immunoblot of the same purified sample, probed with an anti-human IgG HRP-conjugated antibody. Lanes NR and R represent non-reducing and reducing conditions, respectively. Red arrows indicate the molecular weights of the hIGF-1-Fc monomer and dimer, as predicted from the canonical sequence.

The plant-optimized hIGF-1 gene was fused with an Fc tag to facilitate purification via Protein A affinity chromatography. SDS-PAGE analysis revealed a major band at approximately 70 kDa, corresponding to the dimeric form of hIGF-1-Fc (Figure 3a, lane NR). Under reducing conditions with β-mercaptoethanol, a ~35 kDa protein band representing the monomeric form was detected, along with traces of the multimeric forms (Figure 3a, lane R). Therefore, this finding confirms the successful dimer assembly of the Fc fusion protein in the eukaryotic *N. benthamiana* system. The purity of plant-produced hIGF-1-Fc was >90% based on visual inspection of stained gels. To further confirm the identity of the target protein, Western blot analysis was conducted. A strong 70 kDa band was detected under non-reducing conditions, while both 35 kDa and minor 70 kDa bands were observed under reducing conditions (Figure 3b and Supplementary Figure S3). These protein band profiles were consistent with those identified in the SDS-PAGE gel. After one-step purification, approximately 0.01 µg of hIGF-1-Fc protein was obtained per g of *N. benthamiana* leaves.

The apparent molecular weight of hIGF-1-Fc (~70 kDa) on SDS-PAGE gel was slightly higher than the calculated theoretical mass of the unglycosylated dimer (~64 kDa), suggesting the presence of post-translational modifications such as N-glycosylation. To confirm and characterize these modifications, LC-MS-based glycoprofiling was performed.

### 3.3. Glycoprofiling Analysis of Plant-Produced hIGF-1-Fc

**Figure 4:**
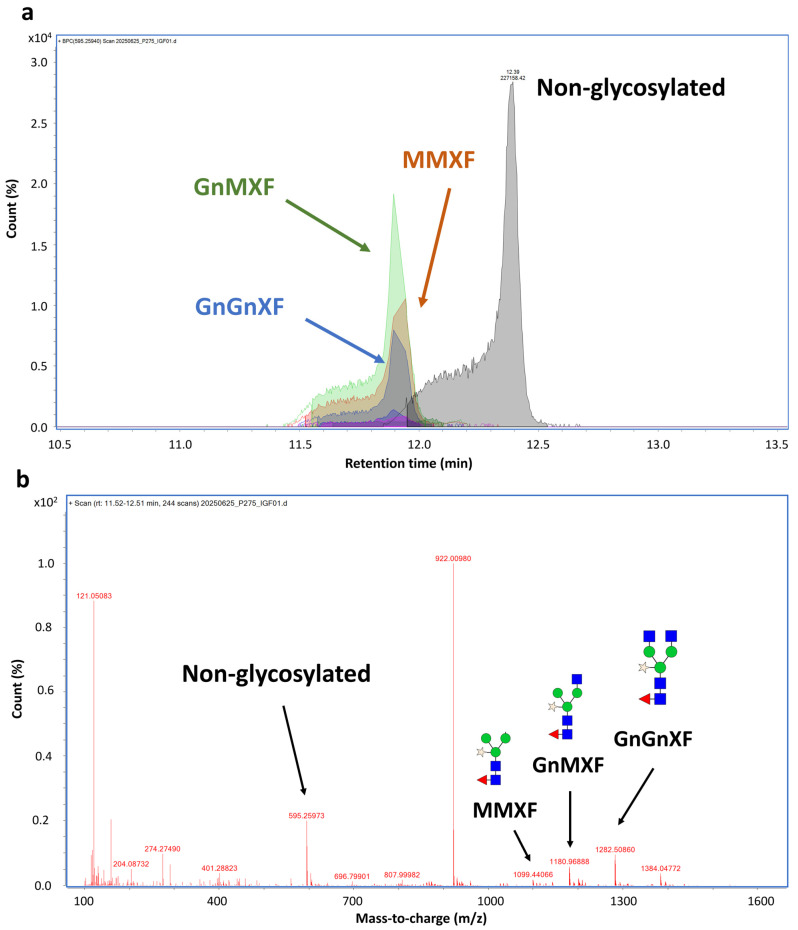
N-glycosylation analysis at N229 position of hIGF-1-FC. Chromatogram of EEQYNSTYR peptide showing peaks of different plant N-glycan attachments (**a**). Non-glycosylated peak was eluted at 12.39 min, while glycosylated peaks were eluted faster between 11.9–12.40 min. MS spectrum of glycosylated mass from 1107–1383 *m*/*z*. The peaks corresponding to GnGnXF, GnMXF, MMXF, and non-glycosylated forms are shown in blue, green, brown, and gray, respectively (**b**). Among 63.1% glycosylated peptides, GnMXF, GnGnXF, and MMXF were major forms of attached N-glycans.

Mass spectrometry analysis confirmed that N-glycosylation was a major post-translational modification contributing to the observed mass shift. From the MS data, multiple glycans were detected at the N229 position, a common glycosylation site in the Fc region. The corresponding peptide, EEQYNSTYR, was found to be attached to different plant-derived glycoforms. The glycosylated species were observed at around 11.5–12.4 min, and its mass difference was calculated for glycan types. Around 63% of the molecule was glycosylated. The glycans detected at this site included GnMXF, GnGnXF, and MMXF (Figure 4 and Supplementary Table S2). Meanwhile, the non-glycosylated peptide was detected at 12.39 min.

### 3.4. Bioactivity of Plant-Produced hIGF-1-Fc in Mammalian Cell Line

**Figure 5:**
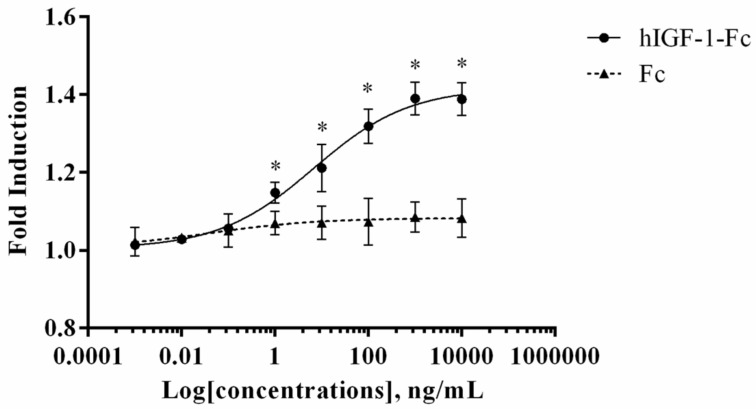
Dose–response curve of plant-produced hIGF-1-Fc fusion protein in a cell proliferation assay. Increased proliferation of MCF-7 cells was observed with hIGF-1-Fc treatment as compared to the Fc fragment control. Experiments were performed in triplicate, and data are expressed as mean ± SD. * *p* < 0.05 indicates statistical significance.

An MTT assay was performed to evaluate the in vitro proliferative activity of plant-produced hIGF-1-Fc in the human breast cancer cell line (MCF-7) and compared to the control Fc fragment. This assay measures the reduction of MTT by metabolically active cells to form insoluble formazan crystals, which are dissolved for absorbance reading to quantify cell proliferation. As shown in Figure 5, treatment with plant-produced hIGF-1-Fc significantly enhanced MCF-7 cell proliferation in a dose-dependent manner (*p* < 0.05) (Supplementary Tables S3 and S5). A notable increase in fold induction was observed at concentrations ranging from 0.001 ng/mL to 10,000 ng/mL. Dose–response analysis yielded an EC_50_ value of approximately 6.765 ng/mL for the plant-derived hIGF-1 fusion protein. These results demonstrated the potency of hIGF-1-Fc in stimulating breast cancer cell proliferation.

**Figure 6:**
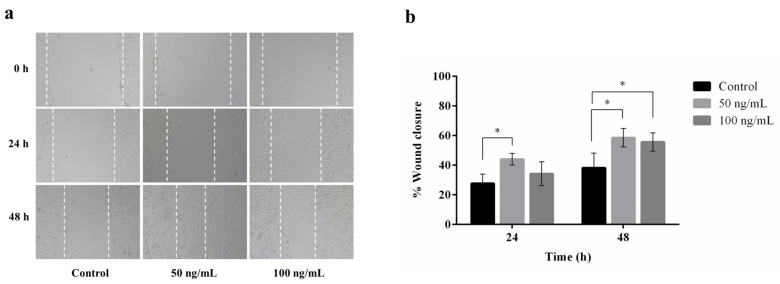
Effect of plant-produced hIGF-1-Fc on NIH3T3 cell migration. Wound healing assays were conducted with hIGF-1-Fc at concentrations of 50 ng/mL and 100 ng/mL. (**a**) Representative images of cell migration at 0 h, 24 h, and 48 h. (**b**) Quantitative analysis of wound closure at different time points. Data are presented as mean ± SD (*n* = 3). * *p* < 0.05 indicates statistical significance.

A wound healing assay was carried out to assess the migratory activity of plant-produced hIGF-1-Fc on mouse embryonic fibroblasts (NIH3T3). This assay stimulates in vitro wound healing closure by creating a linear scratch on a confluent monolayer of cells and monitoring cell migration into the scratched area over time. Treatment with plant-produced hIGF-1-Fc at a concentration of 50 ng/mL significantly promoted NIH3T3 cell migration (Figure 6a), with the maximum cell migration distance observed at 48 h (Figure 6b). Statistical analysis showed a significant improvement in wound closure for the hIGF-1-Fc-treated groups (50 and 100 ng/mL) compared to the untreated control group (0 ng/mL) (*p* < 0.05) (Supplementary Tables S4 and S6). Interestingly, the 50 ng/mL concentration resulted in a higher number of migrating fibroblasts (44.06% wound closure) at 24 h compared to 100 ng/mL (34.25% wound closure). By 48 h, there was no significant difference in cell migration between the 50 ng/mL and 100 ng/mL treatment groups (58.58% vs. 55.63% wound closure).

### 3.5. Proteomics Clustering Analysis

**Figure 7:**
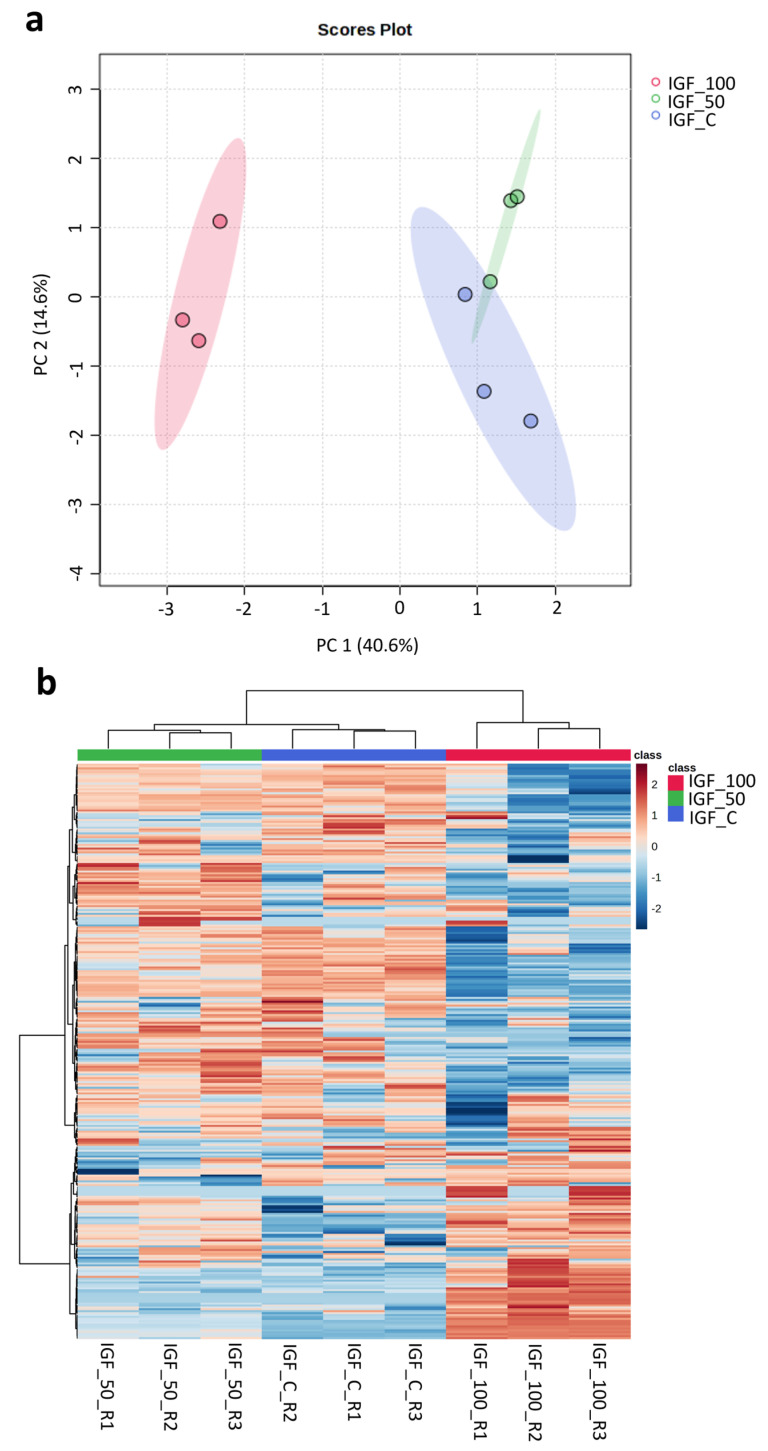
Proteomics analysis displaying the PCA plot and heatmap of proteomes across control and plant-produced hIGF-1-Fc treatments at concentrations of 50 ng/mL and 100 ng/mL. Colors represent treatment groups: red, IGF-100; green, IGF-50; blue, IGF-C. Three biological replicates were analyzed per treatment. (**a**) In the PCA plot, control and IGF_50 samples were clustered close together, but three replicates of IGF_100 were clearly separated apart. The colored ellipses around each sample group represent a 95% confidence interval. (**b**) In the heatmap, control and IGF_50 groups were also clustered together, and IGF_100 group was separated apart. Color gradient indicates protein expression intensity, with red representing high expression and blue representing low expression.

Using mouse NIH3T3 fibroblasts as a model, cells treated with plant-based hIGF-1-Fc at 50 and 100 ng/mL showed differences in cellular protein change from the control. In the principal component analysis (PCA) plot (Figure 7a), hIGF-1-Fc (50 ng/mL)-treated fibroblasts (in green) were slightly separated from the control group (in blue), mainly in the PC2 axis. On the contrary, cells treated with hIGF-1-Fc at 100 ng/mL (in red) exhibited a more pronounced shift from the control along the PC1 direction. The heatmap results (Figure 7b) were well correlated with the PCA data and demonstrated trivial changes between the proteomes of the control and 50 ng/mL hIGF-1-Fc-treated group. However, fibroblasts treated with 100 ng/mL of hIGF-1-Fc displayed obvious proteomic changes, forming a distinct cluster compared to the control group. These results indicate that plant-derived hIGF-1-Fc affected the cellular fibroblast proteome in a dose-dependent manner.

### 3.6. Proteomics Statistical Analysis

Proteome change was determined using one-way ANOVA across three groups. Table 1 shows the significant proteins identified by ANOVA, followed by Tukey’s post hoc analysis. Among 407 protein groups detected, 91 proteins were significantly different proteins across the three groups, and 315 proteins were not significantly different (Supplementary Table S7). Among the 91 significant proteins, a number of proteins with increased intensity in the plant-produced hIGF-1-Fc (100 ng/mL) group were cytoskeleton proteins, responsible for cell scaffolding and structural support. They were, for example, actin, tubulin, profilin, cofilin, actinin, moesin, and elongation factors. Additionally, the levels of proteins related to the energy production and glycolysis pathway were also increased in the 100 ng/mL hIGF-1-Fc treatment. Those were, for example, phosphoglycerate mutase 1, malate dehydrogenase, hydroxyacyl-coenzyme A dehydrogenase, and isocitrate dehydrogenase. In contrast, the levels of several large ribosomal subunit proteins, such as large ribosomal subunit protein uL2, uL4, uL5, uL22, eL8, eL13, eL18, eL28, and eL31, were detected with a lower level in the plant-based hIGF-1-Fc group. Interestingly, the levels of histone proteins seemed to decrease in the hIGF-1-Fc treatment.

### 3.7. GO Function Enrichment and KEGG Pathway Analysis

**Figure 8:**
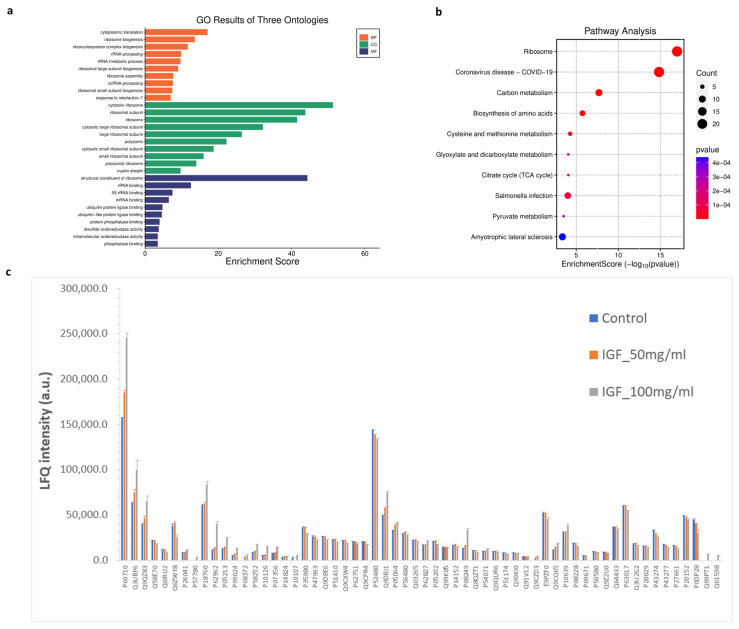
GO and KEGG enrichment analysis of significantly detected proteins in comparisons between the two treatment groups (plant-produced hIGF-1-Fc: 50 and 100 ng/mL) and the untreated control. (**a**) Illustrations of GO-enriched biological processes, cellular components, molecular functions, and (**b**) KEGG enrichment pathway analyses. The color of KEGG terms indicates the *p*-value, and dot size indicates the number of enriched proteins. (**c**) Bar graph presenting average LFQ intensity of significant proteins in separated protein class according to gene ontology.

To explore biological pathways correlated to significant proteins, gene ontology (GO) and Kyoto Encyclopedia of Genes and Genomes (KEGG) pathway enrichment analyses were conducted. The GO analysis identified enrichment in 1257 biological processes, 238 cellular components, and 160 molecular functions. Additionally, KEGG pathway analysis revealed 150 significantly enriched pathways. The most prominent GO terms and KEGG pathways are illustrated in Figure 8. In biological processes (BP), cytoplasmic translation and ribosome biogenesis were the most affected cellular processes by hIGF-1-Fc treatment (Figure 8a). In the molecular functions (MF) and cell complex (CC) enrichment, proteins associated with ribosome, rRNA binding, mRNA binding, ubiquitin protein ligase, and phosphatase binding were enriched. In KEGG analysis, biological pathways related to ribosome, COVID-19, carbon metabolism, biosynthesis of amino acids, cysteine and methionine metabolism, glyoxylate and dicarboxylate metabolism, citrate cycle (TCA cycle), and pyruvate metabolism were pathways related to the proteins changed upon hIGF-1-Fc treatment (Figure 8b). The bar graph in Figure 8c demonstrates the levels of significant proteins across the three groups in a separate protein class. The results imply that ribosomal proteins, ribosomal biogenesis pathways, and carbon metabolism are highly affected by plant-produced hIGF-1-Fc treatment and could be mechanisms correlated to hIGF-1-promoted cell proliferation and would healing.

## 4. Discussion

For several decades, in vitro studies have demonstrated that the hIGF system is a key regulator of cell differentiation, proliferation, and survival [27] hIGF-I, also known as somatomedin C, shares structural similarities with insulin and exhibits significant anabolic effects in humans [28]. Clinically, hIGF-1 has shown therapeutic potential in managing type 1 and 2 diabetes, short stature syndrome, and wound healing [6,29]. Beyond treatment applications, growth factors like hIGF-1 are essential in the cultivated meat industry for promoting mammalian cell growth in bioreactors, ultimately yielding edible meats [2]. In the cosmetics industry, they are incorporated into skincare products to promote skin rejuvenation, collagen production, and improve overall skin health [30,31]. The demand for growth factors across biotechnology, pharmaceutical, and cosmetics sectors continues to grow, yet high production costs limit their accessibility. This study addresses these limitations by using plant-based expression systems as cost-effective bioreactors to produce recombinant hIGF-1 and with the efficacy validated in mammalian cell line models.

Herein, we developed a recombinant hIGF-1 protein conjugated with an Fc fragment at the C-terminus. The fusion protein construct included a flexible (GGGGS)3 Gly-Ser repeat linker between hIGF-1 and Fc [32]. The hIGF-1-Fc was produced in *N. benthamiana* using a plant geminiviral vector. In particular, we adapted the pBYR2e vector in this study, which has been engineered for high-level protein expression via rolling circle replication [15]. SDS-PAGE under non-reduced conditions showed a distinct band at approximately 70 kDa, matching the predicted mass of the hIGF-1-Fc dimer based on the canonical protein sequence. This result indicates successful expression and correct assembly of the plant-derived Fc fusion protein [16]. After reduction, a monomeric form appeared at around 35 kDa, consistent with disulfide bond cleavage. In addition to the main hIGF-1-Fc bands, we observed multiple bands by Western blot analysis that may indicate the presence of different proteoforms or partially reduced dimers. For example, the ~70 kDa band under reducing conditions may result from incomplete disulfide bond reduction [33,34], though this was not further investigated. Moreover, the detection of faint bands above or below the expected molecular weight may suggest higher-order multimers or degradation. These phenomena are common for Fc fusion proteins and can result from partial proteolysis or heterogeneity in PTMs [35,36]. One potential contributing factor is the absence of a protease inhibitor cocktail during extraction, which may have led to partial degradation and formation of additional proteoforms [37,38]. Although downstream purification improved the purity and removed many non-specific bands (Figure 3), a minor ~35 kDa band remained (Supplementary Figure S3), consistent with monomeric hIGF-1-Fc. Altogether, our findings demonstrate that the plant-based system can effectively produce recombinant proteins in their native multimeric state. However, the presence of putative proteoforms and partially reduced species highlights the need for further optimization. A key limitation of this study is the absence of synthetic controls to confirm antibody specificity. Future studies should employ biochemical characterization of these species and optimize extraction protocols, including the use of protease inhibitors and positive or negative controls to improve product integrity and quality.

The expression level of plant-produced hIGF-1-Fc was optimized by evaluating different dpi for harvest. Similar to earlier studies on transient expression of immunoglobulins and vaccine antigens in tobacco, the optimal harvest period was observed between 3 and 6 dpi [39,40,41,42]. For instance, immune checkpoint antibodies in *Nicotiana benthamiana* showed a three-fold-higher yield at 4–6 dpi [39,40]. Likewise, expression of the SARS-CoV nucleocapsid protein peaked at 3 dpi with a final yield of 79 µg/g fresh weight [41]. Prior research also explored the impact of signal peptides on expression yields in plants. Panahi et al. demonstrated that including the bacterial Lam B signal sequence slightly reduced the expression level of pro-IGF-1B (30 ng/mg of total protein) compared to constructs without a signal peptide (36–43 ng/mg of total protein) [42]. Furthermore, expression of growth factor FGF without a signal sequence resulted in a five-fold-higher levels than with a signal peptide, suggesting that the effect of signal peptides may vary depending on protein stability across different cellular compartments [11]. Recombinant human IGF-1 has been widely explored in *E. coli* due to high expression levels, rapid growth, and ease of genetic manipulation. Kim et al. reported the production of IGF-1 fusion protein in *E. coli JM109* (at least 30% of the total bacterial protein) in the form of insoluble inclusion bodies. To purify hIGF-1-Fc, we used protein A affinity chromatography and obtained a purity level of >90%. Post-purification yield reached approximately 0.01 µg/g leaf fresh weight, which is lower than the levels reported for pro-IGF-1B in transgenic plants [42]. However, our transient expression system allowed for faster production at 3 dpi compared to their study in stable expression. The findings of this study complement and expand upon those of Musiychuk et al. [43], who demonstrated the production and biological activity of plant-derived hIGF-1 and other erythropoietic growth factors using a TMV-based expression system. While previous work established the feasibility of using plant platforms for cytokine production and confirmed bioactivity through cell proliferation and differentiation assays, the current study provides additional layers of characterization. Specifically, functional activity was assessed in a wound healing model, LC-MS-based glycoprofiling was employed to define Fc glycoform heterogeneity, and label-free quantitative proteomics was performed on NIH3T3 fibroblasts. Furthermore, the Fc-fusion format achieved a simplified one-step purification process. To our knowledge, hIGF-1-Fc fusion has not previously been produced in plants. Although this study successfully expressed hIGF-1-Fc, more efforts are needed to enhance the yield. Strategies could include alternative promoters, optimizing signal sequences, or using different expression vectors [44,45,46].

The MCF-7 breast cancer cell line was employed in this study to evaluate the cell-proliferation-stimulating effects of plant-produced hIGF-1-Fc. Human IGFs are well-known to promote cellular proliferation by driving cell cycle progression and eliciting antiapoptotic effects [47]. Through binding with the hIGF-1 receptor (hIGF-1R), hIGF-1 activates the PI3K and MAPK pathways, both critical for cell division and survival [48,49]. High levels of hIGF-1 have been strongly associated with an increased risk of breast cancer, suggesting its significant role in cancer development and progression [50,51]. In this case, MCF-7 cells provided a relevant in vitro model to test the bioactivity of plant-derived hIGF-1-Fc. After 48 h of treatment, plant-derived hIGF-1-Fc stimulated MCF-7 cell proliferation in a concentration-dependent manner and resulted in an approximate 1.2-fold increase [47]. This confirmed that the hIGF-1 within the fusion construct is biologically active and effectively promotes MCF-7 cell growth. By contrast, cells treated with the plant-derived Fc alone showed no proliferative effect. These data are in line with previous studies demonstrating the mitogenic role of hIGF-1 on MCF-7 cells. The ginsenoside Rg1 promoted MCF-7 cell proliferation by activating the hIGF-1R pathway [52]. Treatment with 1 pM and 1 µM Rg1 led to 1.33- and 1.55-fold increases in cell number, respectively, after 48 h [49]. Likewise, Rajoria et al. (2023) reported that stimulation with 5 nM hIGF-1 significantly increased glycolytic ATP production in MCF-7L cells, suggesting a metabolic shift toward glycolysis to meet the energy demands of proliferation [50]. Together, these studies corroborate the established role of IGF-1 in promoting MCF-7 cell growth and metabolism and further validate the functionality of our plant-produced hIGF-1-Fc.

Moreover, NIH3T3 fibroblast cells were used as a model to mimic natural skin [53,54] and to assess the wound healing potential of plant-produced hIGF-1-Fc. hIGF-1 is a key factor involved in the tissue repair mechanism through the granulation process [55]. The scratch wound assay demonstrated that plant-based hIGF-1-Fc significantly enhanced NIH3T3 cell migration. At 24 h, the wound area was reduced by 44% with the 50 ng/mL hIGF-1-Fc treatment and further progressed to 58% reduction by 48 h. These findings indicate that the recombinant hIGF-1 retains its biological activity to promote healing. Similar studies have shown that other plant-derived growth factors can also induce wound healing [9,10], which is consistent in the current study. On the contrary, non-treated control cells exhibited only 28–38% wound closure. We selected 50 ng/mL and 100 ng/mL IGF-1 concentrations based on the established literature supporting their relevance in fibroblast-based wound healing assays. These doses effectively stimulate key processes such as proliferation and migration without inducing cytotoxic effects [56,57]. The 100 ng/mL concentration is commonly used as a near-maximal dose to capture the full therapeutic potential of IGF-1, while the 50 ng/mL dose provides insight into sub-maximal effects and allows for a basic dose–response comparison [58]. Importantly, these concentrations fall within the physiologically relevant range observed during wound healing and help avoid non-specific or inhibitory effects that may occur at hyper-physiological levels [59].

In this study, proteomics analysis and GO function enrichment and KEGG pathway analysis were also conducted to discern biological mechanisms in correlation with hIGF-1 treatment. With proteomics analysis, cytoskeleton and ribosomal proteins were significantly changed upon 100 ng/mL hIGF-1 treatment. This correlated with the subsequent enrichment analysis, where biological processes of cytoplasmic translation and ribosome biogenesis were enriched. These processes play major roles in protein synthesis, cell proliferation, differentiation, and apoptosis [60]. Additionally, structural constituents of ribosome, rRNA binding, mRNA binding, and ubiquitin protein ligase binding functions were involving molecular functions. These functions play a vital role in regulating various aspects of wound healing through protein modification and degradation [61]. KEGG analysis revealed that the pathways of ribosome, carbon metabolism, biosynthesis of amino acids, citrate cycle (TCA cycle), and pyruvate metabolism were affected by hIGF-1 treatment. These signaling pathways could be key pathways correlated to the promotion of cell proliferation and wound healing processes [62].

Our hIGF-1 fusion protein includes a human Fc fragment containing a conserved N-glycosylation site at position N229. Glycan profiling revealed that approximately 63% of peptides were glycosylated, with GnMXF, GnGnXF, and MMXF as the major plant-type N-glycans attached [63]. Meanwhile, recombinant hIGF-1 produced in *E. coli* lacks post-translational modifications such as glycosylation, which can influence protein solubility, stability, biological activity, and half-life [29]. Although bacterial expression systems offer speed and high yields, they often require additional refolding steps and lack disulfide bond formation or glycan maturation capabilities [64,65]. In contrast, plant-based systems support disulfide bond formation and N-glycosylation, which allows for production of structurally and functional intact proteins [65]. These host-specific differences may contribute to distinct proteomic responses observed in mammalian cells treated with plant-derived hIGF-1-Fc. While the absence of a commercial hIGF-1 control is a limitation, the inherent functional and structural differences between expression systems justify the biological relevance of our findings.

Relevant proteomics studies using *E. coli*-derived IGF-1 have demonstrated growth-factor-specific modulation of cellular processes. For example, King et al. [13] reported that IGF-1 stimulation of murine C2C12 myoblasts increased the presence of cytoskeletal proteins such as cofilin and Rho-GDI, along with metabolic enzymes including enolase and Hsp70. Similarly, our proteomic analysis in NIH3T3 fibroblasts treated with plant-derived hIGF-1-Fc revealed increased abundance of actin, tubulin, profilin, cofilin, and elongation factors, as well as enzymes involved in glycolysis and energy production (e.g., phosphoglycerate mutase 1, isocitrate dehydrogenase). These results support a conserved role of IGF-1 in promoting cytoskeletal remodeling and metabolic activity. In the study by Nagano et al. [12], IGF-1 treatment of Swiss 3T3 fibroblasts modulated PI3K/AKT signaling and selectively enhanced levels of ribosomal proteins, translation factors, and actomyosin components. Our data similarly indicate that hIGF-1-Fc treatment reduced the production of large ribosomal subunit proteins (e.g., uL2, uL4, eL8), while pathway enrichment analyses highlighted cytoplasmic translation, ribosome biogenesis, and carbon metabolism as significantly affected processes. Together, these findings demonstrate that IGF-1, regardless of production source, consistently alters cytoskeletal and translational pathways across different cell types. The distinct glycosylation and Fc-fusion features of our plant-derived hIGF-1 may contribute to the specific proteomic signature and various proteoforms, as observed in our study. In addition, further bioinformatics analysis using tools such as KEGG, GO, DAVID, and STRING should be further implemented to supply shotgun proteomics analysis, digging deeply into the biological functions and inclusively discerning the proteome changes according to the hIGF-1-Fc protein treatment. The bioinformatics analysis will also assist the outcome of proteoform formation with hIGF-1-Fc treatment.

## 5. Conclusions

A transient expression system in *N. benthamiana* successfully produced a biologically active hIGF-1-Fc fusion protein. This plant-based production platform offers a cost-effective alternative to conventional fermentation methods. The plant-derived hIGF-1-Fc effectively promotes differentiation of breast cancer cells in vitro and enhances wound healing in fibroblasts. Proteomic analysis revealed dose-dependent changes in fibroblast protein expression, significantly affecting cytoskeletal organization, energy metabolism, and ribosomal pathways. At 100 ng/mL, hIGF-1-Fc increased cytoskeletal proteins (actin, tubulin, profilin, cofilin) and metabolic enzymes (phosphoglycerate mutase 1, malate dehydrogenase), indicating enhanced structural remodeling and energy metabolism. Conversely, reduced ribosomal proteins and histones suggest a shift in translational regulation. GO and KEGG pathway analyses further highlighted the enrichment of ribosome biogenesis, carbon metabolism, and amino acid biosynthesis, which may contribute to hIGF-1-induced cell proliferation and wound healing. These findings demonstrate the potential of plant-derived hIGF-1-Fc in modulating key cellular pathways and support its broader application in cell culture and regenerative medicine. Future studies should include direct comparisons with non-plant-derived hIGF-1 to assess efficacy and structural differences, comprehensive proteoform analysis, and mechanistic studies. This research provides a foundation for advancing proteomic insights into plant-based biotherapeutics.

## Figures and Tables

**Table 1:** List of significantly different proteins in comparisons between the two treatment groups (hIGF-1 50 and 100 ng/mL) and the untreated control, analyzed by one-way ANOVA with 0.05 FDR. Three biological samples were analyzed. The symbol “–” indicates the absence of a protein class.

No.	Protein IDs	Protein Names	Gene Names	Protein Class	Razor + Unique Peptides	Sequence Coverage [%]	Score	*p* Value	FDR	Average LFQ Intensity Control	Average LFQ Intensity 50 ng/mL	Average LFQ Intensity 100 ng/mL
**1**	Q8VEK3	Heterogeneous nuclear ribonucleoprotein U	Hnrnpu	-	9	18.5	31.30	6.32 × 10^−10^	1.90 × 10^−7^	0.00	0.00	6752.13
**2**	P99024	Tubulin beta-5 chain	Tubb5	tubulin	18	62.8	170.74	3.82 × 10^−9^	3.83 × 10^−7^	5479.97	6900.93	13,230.00
**3**	P09671	Superoxide dismutase [Mn], mitochondrial	Sod2	oxidoreductase	3	16.7	13.49	3.68 × 10^−9^	3.83 × 10^−7^	4804.80	4814.03	0.00
**4**	P57780	Alpha-actinin-4	Actn4	actin or actin-binding cytoskeletal protein	2	2.4	6.47	1.22 × 10^−8^	9.15 × 10^−7^	0.00	0.00	3033.60
**5**	Q99PT1	Rho GDP-dissociation inhibitor 1	Arhgdia	G-protein modulator	2	20.1	14.63	5.50 × 10^−8^	3.31 × 10^−6^	0.00	0.00	6674.27
**6**	Q9DBJ1	Phosphoglycerate mutase 1	Pgam1	mutase	16	79.9	208.75	8.73 × 10^−8^	4.38 × 10^−6^	49,939.67	57,622.00	74,753.33
**7**	Q61598	Rab GDP dissociation inhibitor beta	Gdi2	G-protein modulator	6	22.9	26.36	1.09 × 10^−7^	4.70 × 10^−6^	0.00	0.00	4951.97
**8**	P08249	Malate dehydrogenase, mitochondrial	Mdh2	dehydrogenase	17	63.6	146.94	2.33 × 10^−7^	8.78 × 10^−6^	12,977.67	16,462.33	33,459.00
**9**	P58252	Elongation factor 2	Eef2	translation elongation factor	39	60.7	321.48	2.90 × 10^−7^	9.69 × 10^−6^	8059.33	9862.90	17,363.67
**10**	P05213	Tubulin alpha-1B chain	Tuba1b	tubulin	15	49.0	95.53	3.68 × 10^−7^	1.0059 × 10^−5^	12,769.00	14,546.67	24,569.67
**11**	P60710	Actin, cytoplasmic 1	Actb	actin and actin-related protein	24	68.0	323.31	3.61 × 10^−7^	1.0059 × 10^−5^	156,963.33	185,090.00	245,403.33
**12**	P62962	Profilin-1	Pfn1	non-motor actin-binding protein	11	91.4	85.66	6.06 × 10^−7^	1.5203 × 10^−5^	11,084.67	14,078.67	39,967.33
**13**	Q01853	Transitional endoplasmic reticulum ATPase	Vcp	primary active transporter	21	41.7	144.96	6.57 × 10^−7^	1.5219 × 10^−5^	6507.83	8583.27	12,508.33
**14**	P68372	Tubulin beta-4B chain	Tubb4b	tubulin	4	57.8	20.09	1.026 × 10^−6^	0.00002206	0.00	3092.93	4930.87
**15**	P63038	60 kDa heat shock protein, mitochondrial	Hspd1	-	23	59.9	227.60	1.62 × 10^−6^	3.2498 × 10^−5^	9488.90	7901.73	13,505.67
**16**	Q61425	Hydroxyacyl-coenzyme A dehydrogenase, mitochondrial	Hadh	-	3	20.1	14.97	0.00000208	3.9129 × 10^−5^	0.00	0.00	5587.93
**17**	Q9CQ65	S-methyl-5-thioadenosine phosphorylase	Mtap	nucleotide kinase	9	46.6	49.67	0.00001644	0.00029109	11,428.33	14,249.33	18,848.67
**18**	P07356	Annexin A2	Anxa2	calcium-binding protein	15	58.4	76.54	2.3959 × 10^−5^	0.00040065	7585.53	8012.63	13,982.67
**19**	Q9D8E6	Large ribosomal subunit protein uL4	Rpl4	ribosomal protein	24	52.5	121.88	4.9777 × 10^−5^	0.00078857	26,020.00	25,327.00	22,445.00
**20**	P05064	Fructose-bisphosphate aldolase A	Aldoa	aldolase	25	81.3	233.20	5.4198 × 10^−5^	0.00081569	32,942.33	38,918.67	41,229.67
**21**	P54071	Isocitrate dehydrogenase [NADP], mitochondrial	Idh2	dehydrogenase	17	43.1	57.47	0.00009511	0.0013632	10,177.17	9926.90	12,328.00
**22**	P08228	Superoxide dismutase [Cu-Zn]	Sod1	oxidoreductase	8	58.4	30.51	0.00012794	0.0016743	18,451.00	17,965.33	14,930.33
**23**	P10126	Elongation factor 1-alpha 1	Eef1a1	translation factor	10	29.7	41.23	0.00012262	0.0016743	4519.57	5741.20	14,470.67
**24**	Q03265	ATP synthase subunit alpha, mitochondrial	Atp5f1a	ATP synthase	29	61.8	290.33	0.0001883	0.0023617	21,643.00	22,408.00	20,415.67
**25**	Q9Z1Q5	Chloride intracellular channel protein 1	Clic1	ion channel	10	52.3	60.80	0.00022731	0.0027368	12,136.00	13,294.67	17,209.67
**26**	P29758	Ornithine aminotransferase, mitochondrial	Oat	transaminase	9	29.2	44.85	0.00028426	0.003169	7568.87	8109.57	6298.13
**27**	P18760	Cofilin-1	Cfl1	non-motor actin-binding protein	13	69.3	162.83	0.00027613	0.003169	60,508.33	61,218.33	83,614.00
**28**	Q9EST5	Acidic leucine-rich nuclear phosphoprotein 32 family member B	Anp32b	chromatin/chromatin-binding, or -regulatory protein	5	21.0	20.71	0.00033516	0.003603	9243.43	9230.03	7850.20
**29**	Q6PAC1	Gelsolin	Gsn	-	23	42.1	181.39	0.00035126	0.0036458	13,712.00	14,174.00	16,514.33
**30**	P10639	Thioredoxin	Txn	oxidoreductase	9	65.7	83.57	0.00037264	0.0037388	30,886.00	30,616.00	37,830.33
**31**	Q05816	Fatty-acid-binding protein 5	Fabp5	transfer/carrier protein	7	54.8	40.05	0.00046673	0.0045318	10,121.67	10,063.17	8700.53
**32**	P35980	Large ribosomal subunit protein eL18	Rpl18	ribosomal protein	7	34.0	52.44	0.00057888	0.0051248	35,643.67	35,527.00	28,636.00
**33**	P62082	Small ribosomal subunit protein eS7	Rps7	ribosomal protein	6	36.1	43.29	0.000578	0.0051248	9635.80	10,517.87	12,125.33
**34**	P52480	Pyruvate kinase PKM	Pkm	kinase	46	76.8	323.31	0.00056453	0.0051248	143,280.00	138,166.67	133,363.33
**35**	Q58E70	Tropomyosin alpha-3 chain	Tpm3	actin-binding motor protein	22	70.2	78.94	0.00060716	0.0052215	21,365.00	21,205.33	17,868.00
**36**	P62908	Small ribosomal subunit protein uS3	Rps3	ribosomal protein	16	73.7	86.90	0.00062987	0.0052664	12,127.00	13,240.33	14,048.67
**37**	Q9CPR4	Large ribosomal subunit protein uL22	Rpl17	ribosomal protein	7	42.4	80.83	0.00075181	0.0061161	20,140.67	19,624.33	17,258.00
**38**	P14152	Malate dehydrogenase, cytoplasmic	Mdh1	dehydrogenase	12	45.2	98.76	0.00081216	0.0062682	16,658.00	17,165.33	15,238.33
**39**	P05202	Aspartate aminotransferase, mitochondrial	Got2	transaminase	23	61.4	172.25	0.0007966	0.0062682	20,688.00	21,339.33	17,041.00
**40**	P51174	Long-chain-specific acyl-CoA dehydrogenase, mitochondrial	Acadl	dehydrogenase	9	24.2	37.13	0.00084378	0.0063495	7992.47	7431.20	6335.93
**41**	P20029	Endoplasmic reticulum chaperone BiP	Hspa5	Hsp70 family chaperone	29	53.7	190.07	0.00092171	0.0067667	15,860.33	15,596.33	13,669.67
**42**	P62827	GTP-binding nuclear protein Ran	Ran	small GTPase	10	46.8	58.25	0.00095812	0.0068665	16,329.33	16,934.67	20,654.33
**43**	P43274	Histone H1.4	H1-4	chromatin/chromatin-binding, or -regulatory protein	15	48.9	53.49	0.0010185	0.0069674	32,338.33	28,665.67	24,969.33
**44**	Q9QZ83	Actin, cytoplasmic 2	Actg1	actin	2	58.5	68.06	0.0010056	0.0069674	39,749.00	46,141.33	64,713.33
**45**	Q9CXW4	Large ribosomal subunit protein uL5	Rpl11	ribosomal protein	8	40.4	45.75	0.0010545	0.0070534	21,779.67	21,250.67	18,601.67
**46**	Q9JJI8	Large ribosomal subunit protein eL38	Rpl38	ribosomal protein	4	50.0	15.51	0.0011868	0.0077656	7474.80	7977.53	10,594.57
**47**	P14824	Annexin A6	Anxa6	calcium-binding protein	8	12.8	16.54	0.0014025	0.0089822	3524.47	3774.50	4491.93
**48**	E9PZF0	Nucleoside diphosphate kinase	Nme1nme2	kinase	16	82.8	86.69	0.0014861	0.0093194	51,947.67	50,911.00	45,294.33
**49**	Q9QUR6	Prolyl endopeptidase	Prep	serine protease	18	36.2	160.87	0.0016692	0.010253	9704.73	10,166.67	8954.80
**50**	Q99K85	Phosphoserine aminotransferase	Psat1	transaminase	19	60.3	134.08	0.0017696	0.010653	14,659.00	14,747.67	13,433.67
**51**	Q3UBP6	Actin, cytoplasmic 1	Actb	actin	1	64.5	21.59	0.0022785	0.013448	62,708.67	74,479.67	99,487.33
**52**	P51410	Large ribosomal subunit protein uL6	Rpl9	ribosomal protein	8	60.4	109.24	0.0025731	0.014894	22,031.33	23,406.00	19,980.00
**53**	P63276	Small ribosomal subunit protein eS17	Rps17	ribosomal protein	7	59.3	65.67	0.0027564	0.015654	10,205.00	10,982.67	11,837.00
**54**	P63017	Heat shock cognate 71 kDa protein	Hspa8	Hsp70 family chaperone	42	61.6	323.31	0.0028242	0.015742	59,902.00	58,445.00	54,160.67
**55**	Q99KI0	Aconitate hydratase, mitochondrial	Aco2	hydratase	13	25.9	76.64	0.0029479	0.016133	8107.70	8037.27	7362.60
**56**	P56480	ATP synthase subunit beta, mitochondrial	Atp5f1b	ATP synthase	25	68.6	294.28	0.0030049	0.016151	29,110.67	30,717.33	28,225.33
**57**	Q6ZWY8	Thymosin beta-10	Tmsb10	actin or actin-binding cytoskeletal protein	3	63.6	17.73	0.0031714	0.016747	37,373.00	40,604.00	25,140.33
**58**	P67984	Large ribosomal subunit protein eL22	Rpl22	ribosomal protein	4	50.8	83.48	0.0033276	0.017269	19,091.67	18,947.00	15,029.67
**59**	P43277	Histone H1.3	H1-3	protease	4	48.4	16.98	0.0036001	0.01806	16,913.33	16,400.00	13,279.00
**60**	Q9Z2U0	Proteasome subunit alpha type-7	Psma7		9	46.4	39.31	0.0035567	0.01806	8670.03	8875.67	7606.10
**61**	P62702	Small ribosomal subunit protein eS4	Rps4x	chromatin/chromatin-binding, or -regulatory protein	16	56.7	96.30	0.0039878	0.019677	16,314.67	17,054.33	15,460.67
**62**	P12970	Large ribosomal subunit protein eL8	Rpl7a	ribosomal protein	14	42.9	95.57	0.0048885	0.023733	19,045.33	20,383.33	17,862.33
**63**	P62918	Large ribosomal subunit protein uL2	Rpl8	ribosomal protein	9	42.4	51.64	0.0056166	0.026835	11,289.67	11,530.67	10,106.47
**64**	P29341	Polyadenylate-binding protein 1	Pabpc1	RNA metabolism protein	4	8.2	17.21	0.0057986	0.027271	4392.00	4259.23	5466.60
**65**	P50580	Proliferation-associated protein 2G4	Pa2g4	protease	18	57.1	113.40	0.00615	0.028479	9321.97	9097.77	8203.57
**66**	P20152	Vimentin	Vim	intermediate filament	41	75.8	323.31	0.0064388	0.029365	49,005.33	46,856.33	44,301.67
**67**	P41105	Large ribosomal subunit protein eL28	Rpl28	ribosomal protein	7	46.0	21.99	0.0066077	0.029685	19,276.67	18,723.00	15,970.67
**68**	P63323	Small ribosomal subunit protein eS12	Rps12	ribosomal protein	4	42.4	31.44	0.0072331	0.032017	6741.43	6526.37	7939.80
**69**	Q9CWJ9	Bifunctional purine biosynthesis protein ATIC	Atic	-	25	66.9	213.45	0.007537	0.032879	14,585.33	14,841.67	11,386.33
**70**	P62900	Large ribosomal subunit protein eL31	Rpl31	ribosomal protein	4	32.8	30.83	0.0080753	0.033759	18,986.33	18,426.00	14,632.67
**71**	P47963	Large ribosomal subunit protein eL13	Rpl13	ribosomal protein	9	41.7	61.51	0.008017	0.033759	26,100.00	25,337.00	22,200.00
**72**	P0DP28	Calmodulin-3	Calm3	calmodulin-related	8	85.2	106.68	0.0079118	0.033759	44,585.00	40,329.00	29,262.00
**73**	P26350	Prothymosin alpha	Ptma	-	5	35.1	93.56	0.00833	0.034347	53,486.67	53,791.67	46,079.00
**74**	P26041	Moesin	Msn	actin or actin-binding cytoskeletal protein	17	35.0	55.42	0.008603	0.034993	8425.43	8195.13	10,952.53
**75**	Q64433	10 kDa heat shock protein, mitochondrial	Hspe1	chaperonin	8	74.5	32.85	0.0088507	0.035521	36,883.00	36,167.33	33,943.67
**76**	P35979	Large ribosomal subunit protein uL11	Rpl12	ribosomal protein	7	59.4	77.63	0.0090699	0.035921	14,956.00	16,245.00	16,556.33
**77**	P10107	Annexin A1	Anxa1	calcium-binding protein	6	26.6	29.55	0.00935	0.03655	2005.67	0.00	4871.20
**78**	P47911	Large ribosomal subunit protein eL6	Rpl6	ribosomal protein	12	41.6	81.45	0.0098371	0.037481	17,518.00	17,086.00	16,377.00
**79**	P47955	Large ribosomal subunit protein P1	Rplp1	ribosomal protein	3	79.8	64.64	0.0097769	0.037481	14,762.33	15,305.33	19,075.33
**80**	P27661	Histone H2AX	H2ax	chromatin/chromatin-binding, or -regulatory protein	3	38.5	54.13	0.010327	0.038856	16,497.00	14,605.33	11,427.43
**81**	P43276	Histone H1.5	H1-5	chromatin/chromatin-binding, or -regulatory protein	7	38.1	18.61	0.010464	0.038884	8272.10	7751.87	7107.23
**82**	E9Q616	AHNAK nucleoprotein (desmoyokin)	Ahnak	-	83	41.8	315.61	0.010628	0.039012	74,622.00	73,850.33	60,056.00
**83**	Q9CZD3	Glycine—tRNA ligase	Gars1	aminoacyl-tRNA synthetase	3	6.3	10.03	0.011634	0.041687	0.00	1983.93	4342.40
**84**	P62751	Large ribosomal subunit protein uL23	Rpl23a	ribosomal protein	8	34.6	34.62	0.011617	0.041687	20,730.33	19,457.33	17,889.67
**85**	Q61699	Heat shock protein 105 kDa	Hsph1	Hsp70 family chaperone	10	19.7	41.66	0.012453	0.043086	3790.20	4263.23	4571.70
**86**	Q91V12	Cytosolic acyl coenzyme A thioester hydrolase	Acot7	transferase	4	13.1	10.93	0.012417	0.043086	3947.23	4232.43	3802.13
**87**	P08030	Adenine phosphoribosyltransferase	Aprt	esterase	7	62.8	48.03	0.012202	0.043086	1133.50	4454.63	8172.63
**88**	P63325	Small ribosomal subunit protein eS10	Rps10	Hsp70 family chaperone	6	35.8	11.38	0.012964	0.043359	8004.17	8093.47	9416.30
**89**	Q6IRU2	Tropomyosin alpha-4 chain	Tpm4	ribosomal protein	16	64.1	92.19	0.012825	0.043359	11,235.30	11,205.00	7898.63
**90**	Q3U2G2	Heat shock 70 kDa protein 4	Hspa4	actin-binding motor protein	29	45.6	223.88	0.012766	0.043359	18,301.33	18,635.67	15,595.67
**91**	Q8QZT1	Acetyl-CoA acetyltransferase, mitochondrial	Acat1	acyltransferase	10	34.4	78.09	0.014524	0.048042	10,216.43	10,280.13	8457.63

## Data Availability

The original contributions presented in this study are included in the article/Supplementary Material. Further inquiries can be directed to the corresponding authors.

## Supplementary Materials

The following supporting information can be downloaded at: https://www.mdpi.com/article/10.3390/proteomes13040059/s1, Figure S1: DNA Sequencing chromatograms for hIGF-1-Fc using reverse (78R) and forward (80F) primers; Figure S2: Raw gel bot of day post-infiltration (dpi) optimization of hIGF-1-FC under non-reducing condition for Figure 2a; Figure S3: Raw gel bot for Figure 3b. Lanes II represent reducing condition. Red arrow indicate the predicted molecular weights of the hIGF-1-Fc monomer; Table S1: The band intensity data on hIGF-1-Fc expression; Table S2: Summary of N-glycosylation data; Table S3: Fold induction of hIGF-1-Fc-treated MCF-7 cells; Table S4: The percentage of wound closure on hIGF-1-Fc-treated NIH3T3 cells; Table S5: Statistical comparison of the proliferation of hIGF-1-Fc-treated MCF-7 cells; Table S6: Statistical comparison of the percentage of wound closure of hIGF-1-Fc-treated NIH3T3 cells; Table S7: Raw proteomics data. This manuscript includes Supplementary Material available for reference. Raw proteomics data can be accessed from JPOST repository, identifier ID: JPST003969.

## References

[1] Rischer H., Szilvay G.R., Oksman-Caldentey K.-M. (2020). Cellular agriculture—Industrial biotechnology for food and materials. Curr. Opin. Biotechnol..

[2] Ahmad S.S., Chun H.J., Ahmad K., Shaikh S., Lim J.H., Ali S., Han S.S., Hur S.J., Sohn J.H., Lee E.J. (2023). The roles of growth factors and hormones in the regulation of muscle satellite cells for cultured meat production. J. Anim. Sci. Technol..

[3] Tripathi N.K., Shrivastava A. (2019). Recent Developments in Bioprocessing of Recombinant Proteins: Expression Hosts and Process Development. Front. Bioeng. Biotechnol..

[4] Venkatesan M., Semper C., Skrivergaard S., Di Leo R., Mesa N., Rasmussen M.K., Young J.F., Therkildsen M., Stogios P.J., Savchenko A. (2022). Recombinant production of growth factors for application in cell culture. iScience.

[5] Bailes J., Soloviev M. (2021). Insulin-Like Growth Factor-1 (IGF-1) and Its Monitoring in Medical Diagnostic and in Sports. Biomolecules.

[6] Laron Z. (2001). Insulin-like growth factor 1 (IGF-1): A growth hormone. Mol. Pathol..

[7] Zhang X., Hu F., Li J., Chen L., Mao Y.-F., Li Q.-B., Nie C.-Y., Lin C., Xiao J. (2024). IGF-1 inhibits inflammation and accelerates angiogenesis via Ras/PI3K/IKK/NF-κB signaling pathways to promote wound healing. Eur. J. Pharm. Sci..

[8] Shanmugaraj B., Bulaon C.J.I., Phoolcharoen W. (2020). Plant Molecular Farming: A Viable Platform for Recombinant Biopharmaceutical Production. Plants.

[9] Bulaon C.J.I., Shanmugaraj B., Oo Y., Rattanapisit K., Chuanasa T., Chaotham C., Phoolcharoen W. (2020). Rapid transient expression of functional human vascular endothelial growth factor in Nicotiana benthamiana and characterization of its biological activity. Biotechnol. Rep..

[10] Hanittinan O., Oo Y., Chaotham C., Rattanapisit K., Shanmugaraj B., Phoolcharoen W. (2020). Expression optimization, purification and in vitro characterization of human epidermal growth factor produced in *Nicotiana benthamiana*. Biotechnol. Rep..

[11] Rattanapisit K., Jantimaporn A., Kaewpungsup P., Shanmugaraj B., Pavasant P., Namdee K., Phoolcharoen W. (2020). Plant-Produced Basic Fibroblast Growth Factor (bFGF) Promotes Cell Proliferation and Collagen Production. Planta Medica Int. Open.

[12] Nagano K., Akpan A., Warnasuriya G., Corless S., Totty N., Yang A., Stein R., Zvelebil M., Stensballe A., Burlingame A. (2012). Functional Proteomic Analysis of Long-term Growth Factor Stimulation and Receptor Tyrosine Kinase Coactivation in Swiss 3T3 Fibroblasts. Mol. Cell. Proteom..

[13] King C.C., Bouic K., Friedmann T. (2009). A fractionation method to identify qauntitative changes in protein expression mediated by IGF-1 on the proteome of murine C2C12 myoblasts. Proteome Sci..

[14] Chen Q., Davis K.R. (2016). The potential of plants as a system for the development and production of human biologics. F1000Research.

[15] Chen Q., He J., Phoolcharoen W., Mason H.S. (2011). Geminiviral vectors based on bean yellow dwarf virus for production of vaccine antigens and monoclonal antibodies in plants. Hum. Vaccines.

[16] Srisangsung T., Phetphoung T., Manopwisedjaroen S., Rattanapisit K., Bulaon C.J.I., Thitithanyanont A., Limprasutr V., Strasser R., Phoolcharoen W. (2024). The impact of N-glycans on the immune response of plant-produced SARS-CoV-2 RBD-Fc proteins. Biotechnol. Rep..

[17] Charnsatabut C., Suwanchaikasem P., Rattanapisit K., Iksen I., Pongrakhananon V., Bulaon C.J.I., Phoolcharoen W. (2025). Optimized expression of human interleukin-15 in Nicotiana benthamiana and in vitro assessment of its activity on human keratinocytes. Biotechnol. Rep..

[18] Benington L., Mo J., Li M., Rajan G., Locher C., Lim L.Y. (2024). In Vitro Assessment of Wound-Healing Efficacy of Stabilized Basic Fibroblast Growth Factor (FGF-2) Solutions. Pharmaceuticals.

[19] Carrera M., Mateos Martín J. (2020). Shotgun Proteomics: Methods and Protocols.

[20] Chambers M.C., Maclean B., Burke R., Amodei D., Ruderman D.L., Neumann S., Gatto L., Fischer B., Pratt B., Egertson J. (2012). A cross-platform toolkit for mass spectrometry and proteomics. Nat. Biotechnol..

[21] Röst H.L., Sachsenberg T., Aiche S., Bielow C., Weisser H., Aicheler F., Andreotti S., Ehrlich H.-C., Gutenbrunner P., Kenar E. (2016). OpenMS: A flexible open-source software platform for mass spectrometry data analysis. Nat. Methods.

[22] Cox J., Mann M. (2008). MaxQuant enables high peptide identification rates, individualized p.p.b.-range mass accuracies and proteome-wide protein quantification. Nat. Biotechnol..

[23] UniProt Consortium (2025). UniProt: The Universal Protein Knowledgebase in 2025. Nucleic Acids Res..

[24] Pang Z., Lu Y., Zhou G., Hui F., Xu L., Viau C., Spigelman A.F., MacDonald P.E., Wishart D.S., Li S. (2024). MetaboAnalyst 6.0: Towards a unified platform for metabolomics data processing, analysis and interpretation. Nucleic Acids Res..

[25] Kanehisa M., Goto S. (2000). KEGG: Kyoto encyclopedia of genes and genomes. Nucleic Acids Res..

[26] Tang D., Chen M., Huang X., Zhang G., Zeng L., Zhang G., Wu S., Wang Y. (2023). SRplot: A free online platform for data visualization and graphing. PLoS ONE.

[27] Salmon W.D., Daughaday W.H. (1957). A hormonally controlled serum factor which stimulates sulfate incorporation by cartilage in vitro. J. Lab. Clin. Med..

[28] Rinderknecht E., Humbel R.E. (1978). The amino acid sequence of human insulin-like growth factor I and its structural homology with proinsulin. J. Biol. Chem..

[29] Iranpoor H., Omidinia E., Vatankhah V., Gharanjik V., Shahbazi M. (2015). Expression of Recombinant Human Insulin-like Growth Factor Type 1 (rhIGF-1) in *Escherichia coli*. Avicenna J. Med. Biotechnol..

[30] Miller-Kobisher B., Suárez-Vega D.V., Velazco de Maldonado G.J. (2021). Epidermal Growth Factor in Aesthetics and Regenerative Medicine: Systematic Review. J. Cutan. Aesthet. Surg..

[31] Quinlan D.J., Ghanem A.M., Hassan H. (2023). Topical growth factor preparations for facial skin rejuvenation: A systematic review. J. Cosmet. Dermatol..

[32] Lin J., Asai S., Selicharová I., Mitrová K., Kaminský J., Young E., Jiráček J. (2023). Recombinant Insulin-Like Growth Factor 1 Dimers: Receptor Binding Affinities and Activation Abilities. Int. J. Pept. Res. Ther..

[33] Chang J.-Y. (1997). A Two-Stage Mechanism for the Reductive Unfolding of Disulfide-containing Proteins. J. Biol. Chem..

[34] Roszkowski M., Mansuy I.M. (2021). High Efficiency RNA Extraction From Sperm Cells Using Guanidinium Thiocyanate Supplemented With Tris(2-Carboxyethyl)Phosphine. Front. Cell Dev. Biol..

[35] Li X., Wang F., Xu W., May K., Richardson D., Liu H. (2013). Disulfide bond assignment of an IgG1 monoclonal antibody by LC-MS with post-column partial reduction. Anal. Biochem..

[36] Natesan R., Dykstra A.B., Banerjee A., Agrawal N.J. (2023). Heterogeneity in Disulfide Bond Reduction in IgG1 Antibodies Is Governed by Solvent Accessibility of the Cysteines. Antibodies.

[37] Clemente M., Corigliano M.G., Pariani S.A., Sánchez-López E.F., Sander V.A., Ramos-Duarte V.A. (2019). Plant Serine Protease Inhibitors: Biotechnology Application in Agriculture and Molecular Farming. Int. J. Mol. Sci..

[38] Ma J., Ding X., Li Z., Wang S. (2021). Co-expression With Replicating Vector Overcoming Competitive Effects Derived by a Companion Protease Inhibitor in Plants. Front. Plant Sci..

[39] Phakham T., Bulaon C.J.I., Khorattanakulchai N., Shanmugaraj B., Buranapraditkun S., Boonkrai C., Sooksai S., Hirankarn N., Abe Y., Strasser R. (2021). Functional Characterization of Pembrolizumab Produced in Nicotiana benthamiana Using a Rapid Transient Expression System. Front. Plant Sci..

[40] Rattanapisit K., Bulaon C.J.I., Strasser R., Sun H., Phoolcharoen W. (2023). In vitro and in vivo studies of plant-produced Atezolizumab as a potential immunotherapeutic antibody. Sci. Rep..

[41] Zheng N., Xia R., Yang C., Yin B., Li Y., Duan C., Liang L., Guo H., Xie Q. (2009). Boosted expression of the SARS-CoV nucleocapsid protein in tobacco and its immunogenicity in mice. Vaccine.

[42] Panahi M., Cheng X., Alli Z., Sardana R., Callaghan M., Phipps J., Altosaar I. (2003). Plant-derived recombinant human insulin-like growth factor precursor prohormone IGF-1B caused differentiation of human neuroblastoma cell lines SH-SY5Y. Mol. Breed..

[43] Musiychuk K., Sivalenka R., Jaje J., Bi H., Flores R., Shaw B., Jones R.M., Golovina T., Schnipper J., Khandker L. (2013). Plant-produced human recombinant erythropoietic growth factors support erythroid differentiation in vitro. Stem Cells Dev..

[44] Villao-Uzho L., Chávez-Navarrete T., Pacheco-Coello R., Sánchez-Timm E., Santos-Ordóñez E. (2023). Plant Promoters: Their Identification, Characterization, and Role in Gene Regulation. Genes.

[45] Rozov S.M., Deineko E.V. (2022). Increasing the Efficiency of the Accumulation of Recombinant Proteins in Plant Cells: The Role of Transport Signal Peptides. Plants.

[46] Nosaki S., Hoshikawa K., Ezura H., Miura K. (2021). Transient protein expression systems in plants and their applications. Plant Biotechnol. (Tokyo).

[47] Dupont J., Le Roith D. (2001). Insulin-like growth factor 1 and oestradiol promote cell proliferation of MCF-7 breast cancer cells: New insights into their synergistic effects. Mol. Pathol..

[48] Vanhaesebroeck B., Alessi D.R. (2000). The PI3K-PDK1 connection: More than just a road to PKB. Biochem. J..

[49] Skolnik E., Lee C., Batzer A., Vicentini L., Zhou M., Daly R., Myers M., Backer J., Ullrich A., White M. (1993). The SH2/SH3 domain-containing protein GRB2 interacts with tyrosine-phosphorylated IRS1 and Shc: Implications for insulin control of ras signalling. EMBO J..

[50] Yee D. (1994). The insulin-like growth factor system as a target in breast cancer. Breast Cancer Res. Treat..

[51] Christopoulos P.F., Msaouel P., Koutsilieris M. (2015). The role of the insulin-like growth factor-1 system in breast cancer. Mol. Cancer.

[52] Chen W.F., Lau W.S., Cheung P.Y., Guo D.A., Wong M.S. (2006). Activation of Insulin-Like Growth Factor I Receptor-Mediated Pathway by Ginsenoside Rg1. Br. J. Pharmacol..

[53] Han H., Hong H., Park S.M., Kim D. (2020). Metal–Electrolyte Solution Dual-Mode Electrospinning Process for In Situ Fabrication of Electrospun Bilayer Membrane. Adv. Mater. Interfaces.

[54] Li X.-J., Huang F.-Z., Wan Y., Li Y.-S., Zhang W.K., Xi Y., Tian G.-H., Tang H.-B. (2018). Lipopolysaccharide Stimulated the Migration of NIH3T3 Cells Through a Positive Feedback Between β-Catenin and COX-2. Front. Pharmacol..

[55] Achar R.A., Silva T.C., Achar E., Martines R.B., Machado J.L. (2014). Use of insulin-like growth factor in the healing of open wounds in diabetic and non-diabetic rats. Acta Cir. Bras..

[56] Garoufalia Z., Papadopetraki A., Karatza E., Vardakostas D., Philippou A., Kouraklis G., Mantas D. (2021). Insulin-like growth factor-I and wound healing, a potential answer to non-healing wounds: A systematic review of the literature and future perspectives. Biomed. Rep..

[57] Yang L., Li X., Zhang S., Song J., Zhu T. (2019). Baicalein inhibits proliferation and collagen synthesis of mice fibroblast cell line NIH/3T3 by regulation of miR-9/insulin-like growth factor-1 axis. Artif. Cells Nanomed. Biotechnol..

[58] Huang Y.-L., Qiu R.-F., Mai W.-Y., Kuang J., Cai X.-Y., Dong Y.-G., Hu Y.-Z., Song Y.-B., Cai A.-P., Jiang Z.-G. (2012). Effects of insulin-like growth factor-1 on the properties of mesenchymal stem cells in vitro. J. Zhejiang Univ. Sci. B.

[59] Lin M.J., Lu M.C., Chang H.Y. (2021). Sustained Release of Insulin-Like Growth Factor-1 from Bombyx mori L. Silk Fibroin Delivery for Diabetic Wound Therapy. Int. J. Mol. Sci..

[60] Jiao L., Liu Y., Yu X.-Y., Pan X., Zhang Y., Tu J., Song Y.-H., Li Y. (2023). Ribosome biogenesis in disease: New players and therapeutic targets. Signal Transduct. Target. Ther..

[61] Zhang Q., Gu R., Dai Y., Chen J., Ye P., Zhu H., He W., Nie X. (2025). Molecular mechanisms of ubiquitination in wound healing. Biochem. Pharmacol..

[62] Wang Z., Zhao F., Xu C., Zhang Q., Ren H., Huang X., He C., Ma J., Wang Z. (2024). Metabolic reprogramming in skin wound healing. Burn. Trauma.

[63] Strasser R. (2022). Recent Developments in Deciphering the Biological Role of Plant Complex N-Glycans. Front. Plant Sci..

[64] Ranjbari J., Babaeipour V., Vahidi H., Moghimi H., Mofid M.R., Namvaran M.M., Jafari S. (2015). Enhanced Production of Insulin-like Growth Factor I Protein in *Escherichia coli* by Optimization of Five Key Factors. Iran. J. Pharm. Res..

[65] Rozov S.M., Permyakova N.V., Deineko E.V. (2018). Main Strategies of Plant Expression System Glycoengineering for Producing Humanized Recombinant Pharmaceutical Proteins. Biochemistry.

