# Supplementary material for: Proteomic Analysis of Plant-Derived hIGF-1-Fc Reveals Proteome Abundance Changes Associated with Wound Healing and Cell Proliferation
Source: Proteomes. 2025 Nov 7;13(4):59. doi: 10.3390/proteomes13040059 (PMC12641928; doi:10.3390/proteomes13040059)

Supplementary material

Supplementary material 1

Raw data of day optimization on hIGF-1-Fc production

Table S1: The band intensity data on hIGF-1-Fc expression

dpi	#1	#2	#3	Average	SD
1	1.60	8.497	10.32	6.80	3.76
3	105.87	75.73	110.28	97.29	15.35
5	91.40	94.82	104.79	97.00	5.68
7	40.21	44.35	77.676	54.08	16.77

Supplementary material 2

Table S2: Summary of N-glycosylation data

N-Glycans	Glycan size (Da)	m/z	Charge	Retention Time (min)	Intensity	%
None	-	595.25945	+2	12.39	227,158.42	36.90
GnGnXF	1576.576141	1383.547521	+2	11.89	63,515.51	10.32
GnMXF	1373.496767	1282.007834	+2	11.89	156,763.63	25.46
GnM3	1095.396597	1142.957749	+2	12.40	9,130.08	1.48
GnM3X	1227.438857	1208.978879	+2	12.27	9,950.31	1.62
MMX	1024.359483	1107.439192	+2	11.95	11,462.47	1.86
MMXF	1170.417393	1180.468147	+2	11.94	105,309.81	17.11
Man5	1216.422873	1203.470887	+2	12.06	11,257.33	1.83
Man6	1378.475698	1284.497299	+2	11.89	13,934.44	2.26

Supplementary material 3

Raw data of the proliferative activity of MCF-7 cells treated with different doses of an hIGF-1-Fc fusion protein in a dose-response experiment.

Table S3: Fold induction of hIGF-1-Fc-treated MCF-7 cells

Group	Concentration (ng/mL)	#1	#2	#3	Average	SD
hIGF-1-Fc	0	1.000	1.000	1.000	1.000	0.00
	0.001	1.011	1.013	1.016	1.013	0.00
	0.01	1.021	1.028	1.035	1.028	0.01
	0.1	1.046	1.059	1.064	1.056	0.01
	1	1.161	1.118	1.166	1.148	0.02
	10	1.277	1.158	1.200	1.211	0.05
	100	1.366	1.312	1.278	1.319	0.04
	1,000	1.428	1.345	1.398	1.390	0.03
	10,000	1.437	1.365	1.363	1.389	0.03
Fc	0	1.000	1.000	1.000	1.000	0.00
	0.001	0.996	1.064	1.006	1.022	0.03
	0.01	1.026	1.028	1.044	1.032	0.01
	0.1	1.027	1.100	1.025	1.050	0.03
	1	1.056	1.105	1.048	1.070	0.02
	10	1.038	1.119	1.056	1.071	0.03
	100	1.057	1.139	1.023	1.073	0.05
	1,000	1.048	1.125	1.083	1.086	0.03
	10,000	1.029	1.125	1.094	1.083	0.04

Supplementary material 4

Raw data of wound healing assay to assess the effect of hIGF-1-Fc treatment on mouse fibroblast NIH3T3 cell line

Table S4: The percentage of wound closure on hIGF-1-Fc-treated NIH3T3 cells

Time (h)	Group	#1	#2	#3	Average	SD
24	Control	20.99	28.31	33.56	27.62	6.31
	50 ng/mL	47.72	44.59	39.86	44.06	3.96
	100 ng/mL	35.27	25.65	41.82	34.25	8.13
48	Control	27.50	39.86	47.18	38.18	9.95
	50 ng/mL	52.50	58.36	64.87	58.58	6.19
	100 ng/mL	56.46	49.05	61.38	55.63	6.21

Supplementary material 5

Statistical analysis of MCF-7 cells proliferation following treatment with hIGF-1-Fc was performed using an unpaired t-test. Data are presented as mean ± SD.

Table S5: Statistical comparison of the proliferation of hIGF-1-Fc-treated MCF-7 cells

Concentration (ng/mL)	Fold induction ± SD	P-values were compared between the hIGF-1-Fc and Fc groups
0	1.000 ± 0.00	-
0.001	1.013 ± 0.00	0.6308
0.01	1.028 ± 0.01	0.6499
0.1	1.056 ± 0.01	0.7589
1	1.148 ± 0.02	0.0088*
10	1.211 ± 0.05	0.0142*
100	1.319 ± 0.04	0.0026*
1,000	1.390 ± 0.03	0.0002*
10,000	1.389 ± 0.03	0.0004*

* $p < 0.05$

Supplementary material 6

Statistical analysis of wound healing assay was performed using two-way ANOVA with Turkey’s test. Data are shown as mean ± SD.

Table S6: Statistical comparison of the percentage of wound closure of hIGF-1-Fc-treated NIH3T3 cells

**Time (h)**	**Group**	**%wound closure**	**P-values (control vs. treated)**	**P-values (50 ng/mL vs. 100 ng/mL)**
24	Control	27.62 ± 6.31	-	-
	50 ng/mL	44.06 ± 3.96	0.0356*	-
	100 ng/mL	34.25 ± 8.13	0.5019	0.2427
48	Control	38.18 ± 9.95	-	-
	50 ng/mL	58.58 ± 6.19	0.0104*	-
	100 ng/mL	55.63 ± 6.21	0.0260*	0.8667

* $p < 0.05$

Supplementary Figure S1: DNA Sequencing chromatograms for hIGF-1-Fc using reverse (78R) and forward (80F) primers.

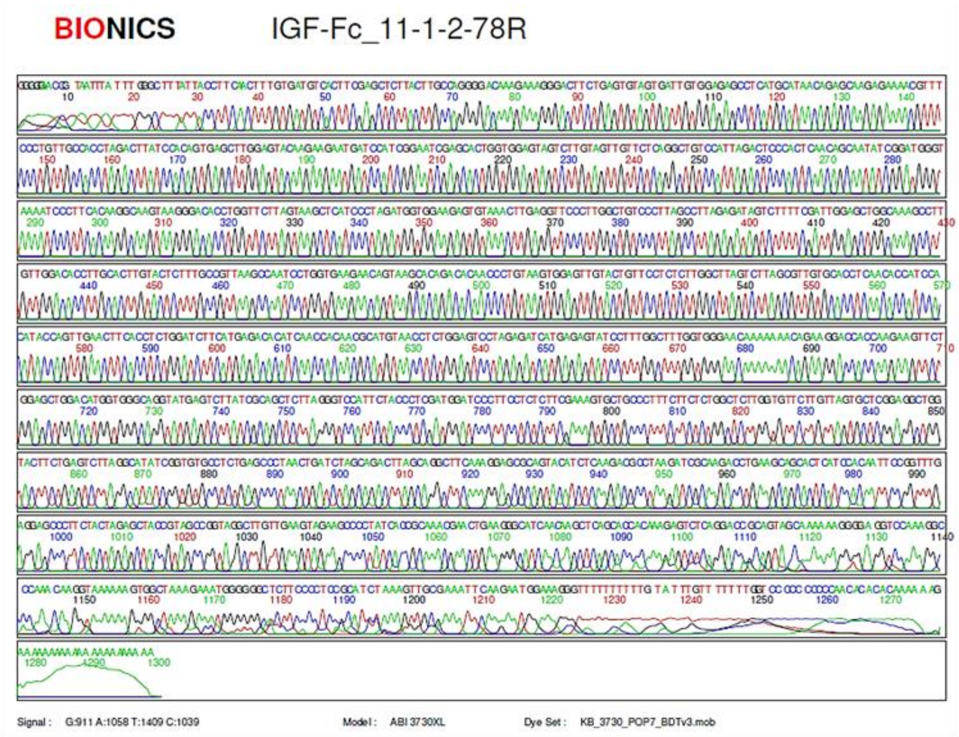

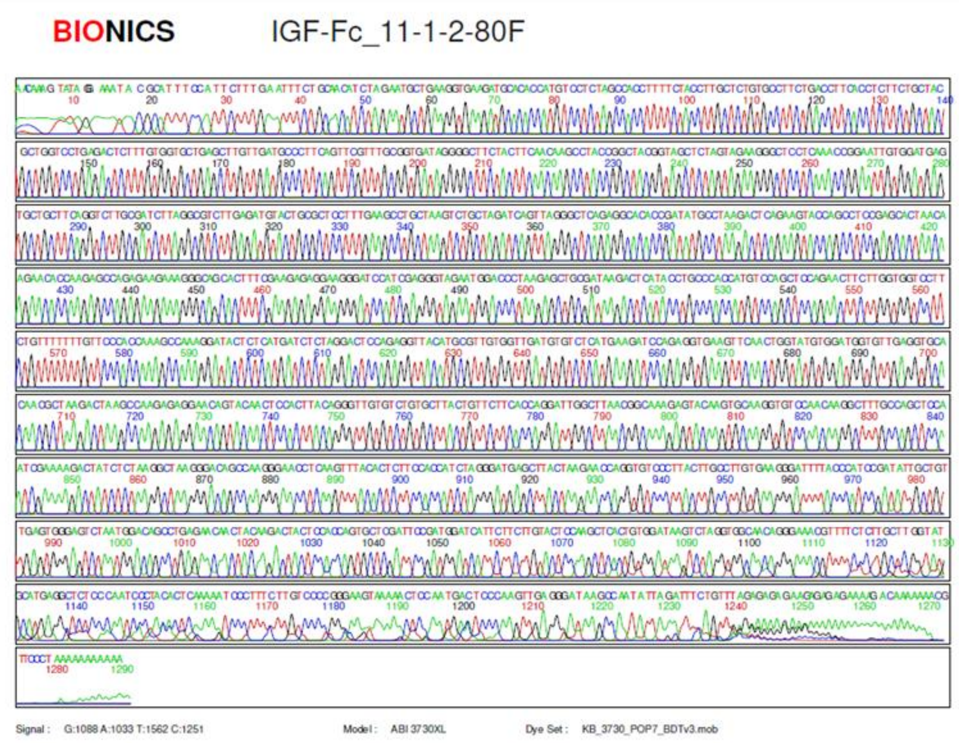

Supplementary Figure S2: Raw gel bot for Figure 2a.

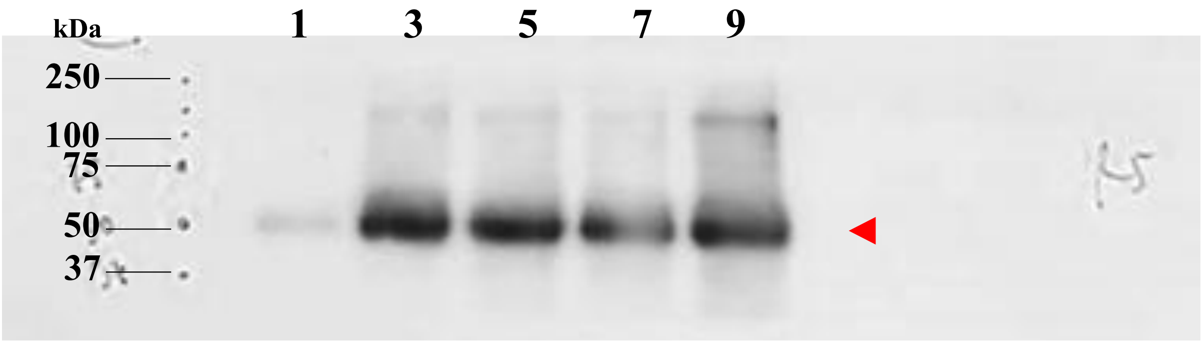

Supplementary Figure S3: Raw gel bot for Figure 3b. Lanes II represent reducing condition. Red arrow indicate the expected molecular weights of the hIGF-1-Fc monomer.

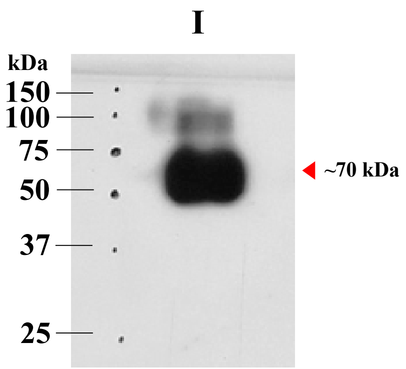

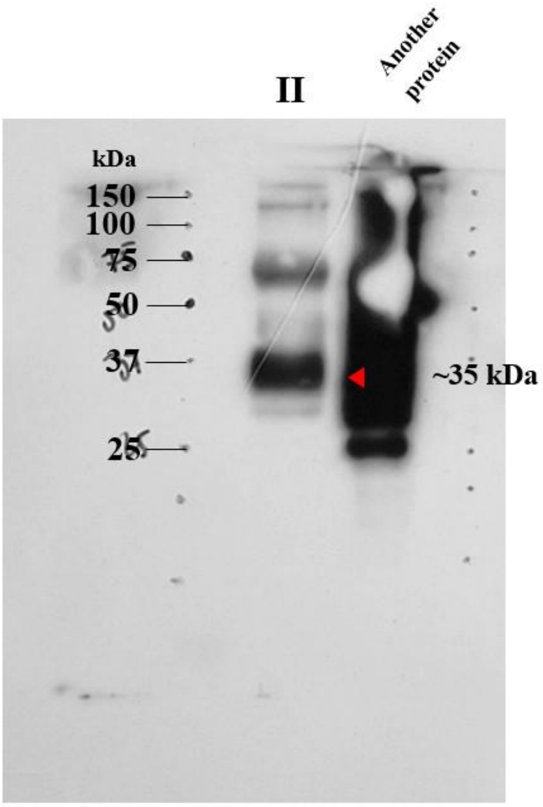

Supplement: Supplementary file 1 [file proteomes-13-00059-s001.zip › Supplementary material.pdf]
